# Recent approaches in the organocatalytic synthesis of pyrroles

**DOI:** 10.1039/d1ra01690c

**Published:** 2021-04-13

**Authors:** Biplob Borah, Kartikey Dhar Dwivedi, L. Raju Chowhan

**Affiliations:** School of Applied Material Sciences, Centre for Applied Chemistry, Central University of Gujarat Gandhinagar 382030 India rchowhan@cug.ac.in

## Abstract

Organocatalysis has emerged as one of the most important tools for the synthesis of diverse structural scaffolds, and has become one of the most important hot topics of current research. Construction of the pyrrole ring has gained much attention from the last few decades due to its remarkable biological activities, pharmaceutical application, intermediate in the synthesis of many natural products, and material science application. With access to these 5-membered aza heterocycles, organocatalytic approaches have provided a new alternative from the perspective of synthetic efficiency, as well as from the green chemistry point of view, and a vast array of synthetic procedures has been developed. Enlightened by the significance of this growing research area, we aim to describe the recent organocatalytic approaches developed for the construction of pyrroles, and organized them based on substrates employed.

## Abstract 2

Recent advances for the synthesis of pyrroles by employing organocatalysis are summarized in this review article.

## Graphical abstract

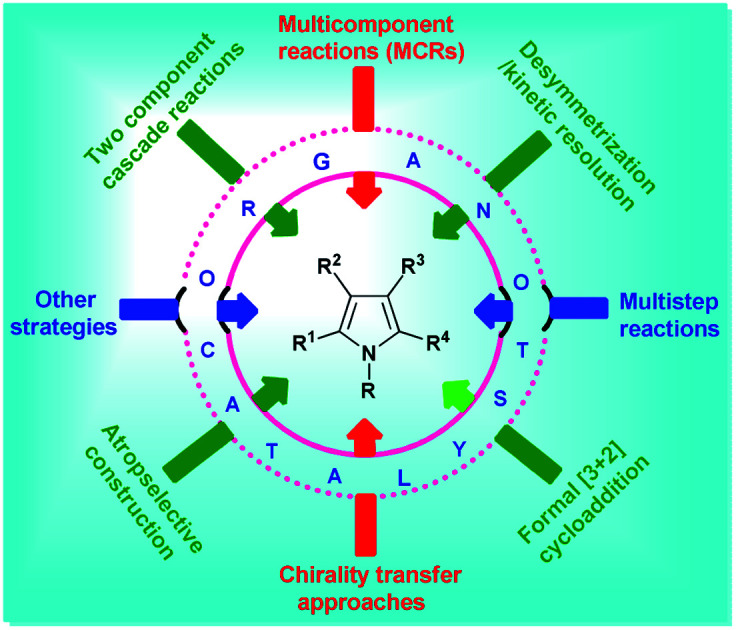

## 1. Introduction

**Fig. 1:**
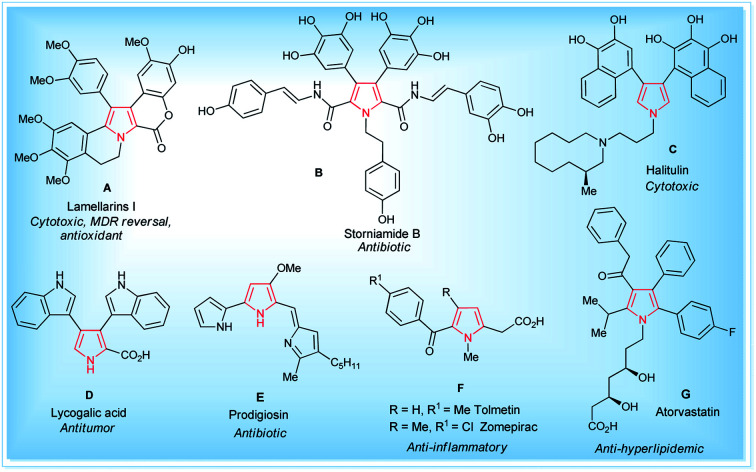
Some natural (A–E)^1,2^ and non-natural compounds (F–G)^3,4^ with biological activity containing the pyrrole moiety.

Pyrroles are the most well-known five-membered nitrogen-containing heterocyclic aromatic compounds, and are the key structural unit of heme and related porphinoid co-factors,^1^ such as heme b, chlorophyll a, vitamin B_12_, and factor 430. Besides these, the pyrrole ring commonly exists in marine natural products,^2^ non-natural products,^3^ drug candidates,^4^ synthetic intermediates,^5^ and optoelectronic materials,^1*b*^ and plays a significant role in the field of medicinal and pharmaceutical chemistry because of their wide-ranging biological activities^6^ (Fig. 1). These tremendous biological activities, pharmaceutical applications, use as a synthetic intermediate in many natural products synthesis and material science application have stimulated interest in the synthesis of pyrroles starting from a traditional one, such as the Hantzsch pyrrole synthesis,^7^ van Leusen,^8^ Knorr,^9^ Paal–Knorr pyrrole synthesis^10^ to non-classical one,^11^ and vast arrays of the synthetic pathway have been developed.

**Fig. 2:**
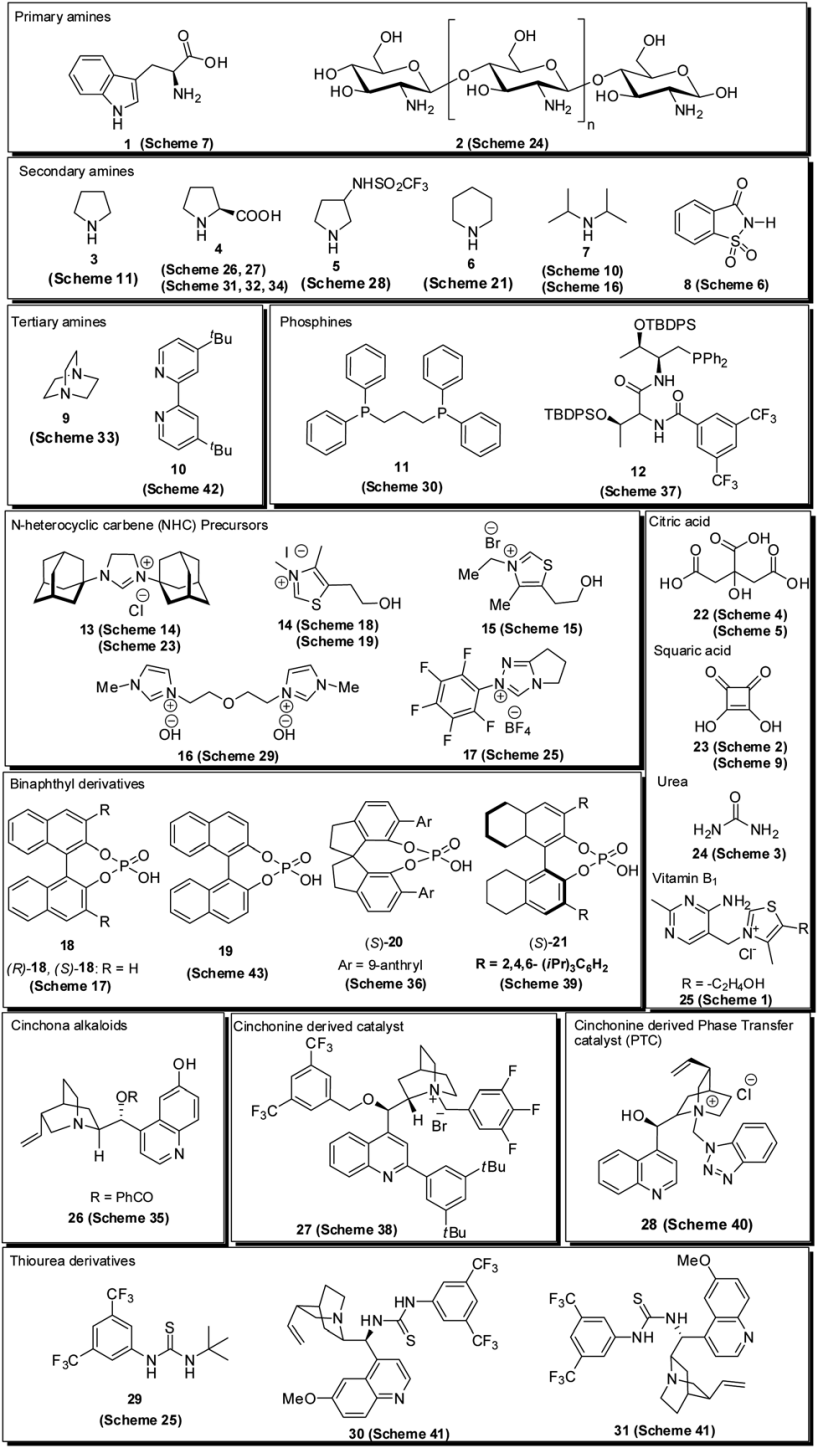
Organocatalyst used for the synthesis of pyrroles.

Over the last decade, the use of small organic molecules called organocatalysts in organic transformation has received increased attention^12^ due to their remarkable properties, including high stability, lower activation energy, high efficiency, transition metal-free nature, reduced toxicity, cost-effectiveness, ready availability and easy recoverability; avoiding expensive catalysts, simple handling in reaction, and the possibility of performing reactions through different activation modes.^13^ In addition, the utilization of chiral organic molecules has emerged as a new platform for the synthesis of enantiomerically enriched compounds.^14^ Various types of organocatalysts employed for the synthesis of pyrroles are listed in Fig. 2. Encouraged by the growth in the area of organocatalysis in organic transformation and the increased application of the pyrrole heterocycle in many branches of chemistry, an interest was born in our mind to highlight the recent developments for the synthesis of pyrroles by systematically using the different organocatalytic systems in this review. Although several reviews have covered the synthesis of pyrroles based on multicomponent reactions,^15^ metal-catalyzed syntheses,^16^ and others,^17^ the organocatalytic approaches toward its synthesis have not been covered with all details until now. This current review aims to provide access to the works on the synthesis of pyrroles by using various organocatalytic strategies and their development to the present state. On behalf of the appropriate understanding and a convenient presentation, the article is classified according to the nature of the substrates used.

## 2. Synthesis of pyrroles by two-component cascade reactions

### 2.1 From dicarbonyl compounds and amines

**Scheme 1:**
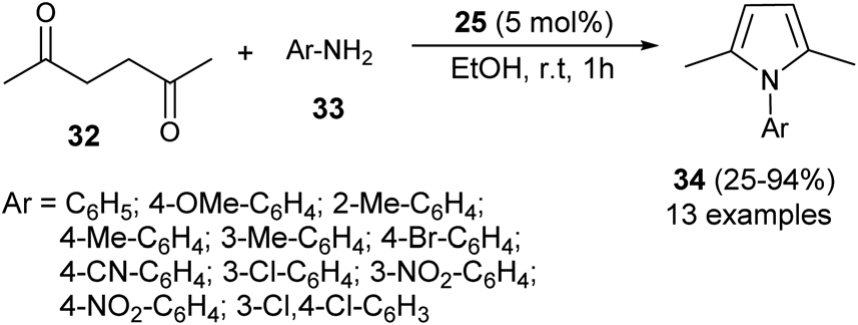
Vitamin B_1_-catalyzed synthesis of substituted pyrroles 34.

In 2012, Darabi *et al.* discovered a practical eco-friendly method for the Paal–Knorr pyrrole synthesis based on the metal-free catalyst (Scheme 1).^18^ Treatment of hexane-2,5-dione 32 with several substituted aromatic amines 33 in ethanol in the presence of vitamin B_1_ (25) as an organocatalyst at room temperature for 1 hour gave the corresponding *N*-substituted pyrroles 34 in moderate to excellent yield (25–94%). Aromatic amines possessing different electron-withdrawing and electron-donating substituents at the C-2, C-3, and C-4 position could react with hexane-2,5-dione smoothly to give the desired product in high yield. However, amines possessing substitution at the C-2 position by the –NO_2_ group had a detrimental effect on the reactivity, and the desired product was not formed due to the existence of the steric hindrance.

**Scheme 2:**
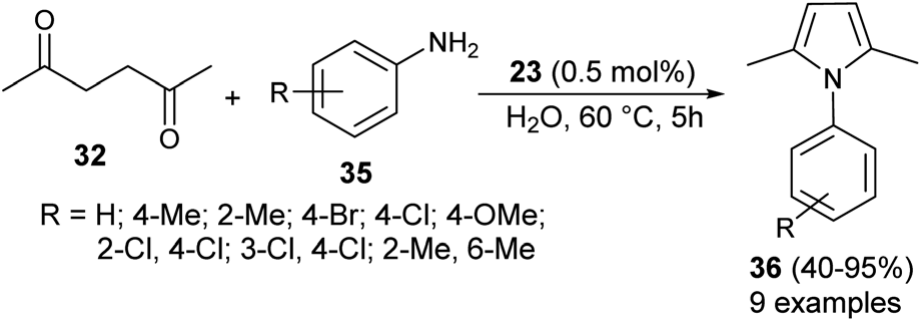
Squaric acid-catalyzed synthesis of pyrrole derivatives 36.

In 2013, Azizi *et al.*^19^ reported a novel two-component strategy that affords *N*-substituted pyrroles 36 from the reaction of hexane-2,5-dione 32 with several aromatic amines 35 in water by introducing squaric acid 23 as an organocatalyst at 60 °C for 5 hours in 40–95% yields (Scheme 2). The reaction performed under the ultrasound irradiation condition also afforded the product in good yield. Although the role of squaric acid 23 in this transformation is not clear, it was believed that the Brønsted acidity is the main reason for which the reaction has proceeded.

**Scheme 3:**
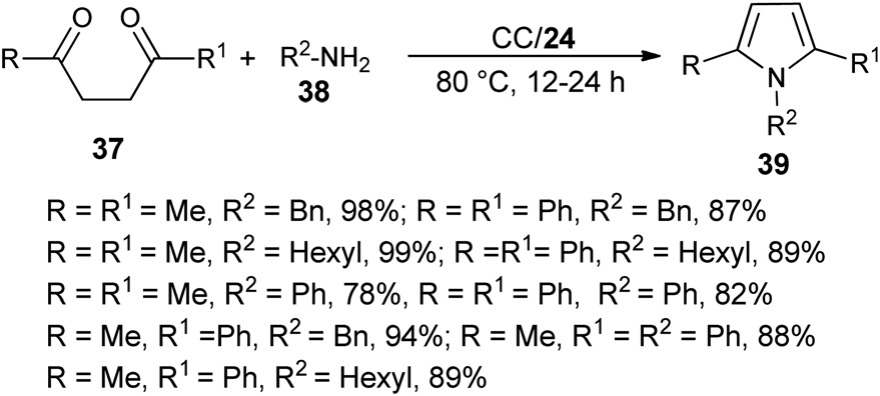
Synthesis of pyrroles 39 from 1,4-diones and amines.

The combination of urea as an organocatalyst with choline chloride (CC) provided an effective solvent/catalyst system for several organic transformations. In this context, Handy and Lavender in 2013 demonstrated an environmentally friendly protocol for the synthesis of *N*-substituted pyrroles 39 in 56–99% yield *via* the reaction of 1,4-diones 37 with several amines 38 in the presence of choline chloride/urea (24) at 80 °C for 12–24 hours (Scheme 3).^20^ In this reaction, the use of urea as an organocatalyst activates the carbonyl compound for the Paal–Knorr cycloaddition reaction with amine by forming two H-bonds with the carbonyl oxygen. Substitution of different alkyl groups in 1,4-dione 37 and amines 38 leads to a wide-ranging substrate scope with high yields.

**Scheme 4:**
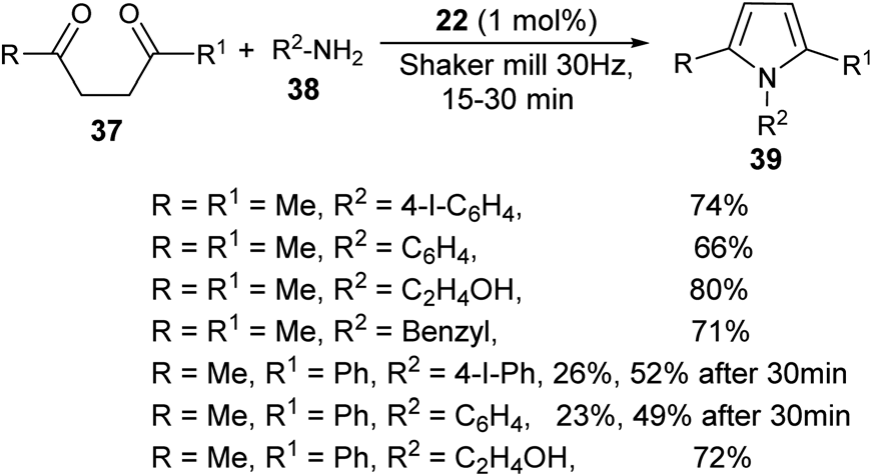
Mechanochemical method for the synthesis of pyrroles.

**Scheme 5:**
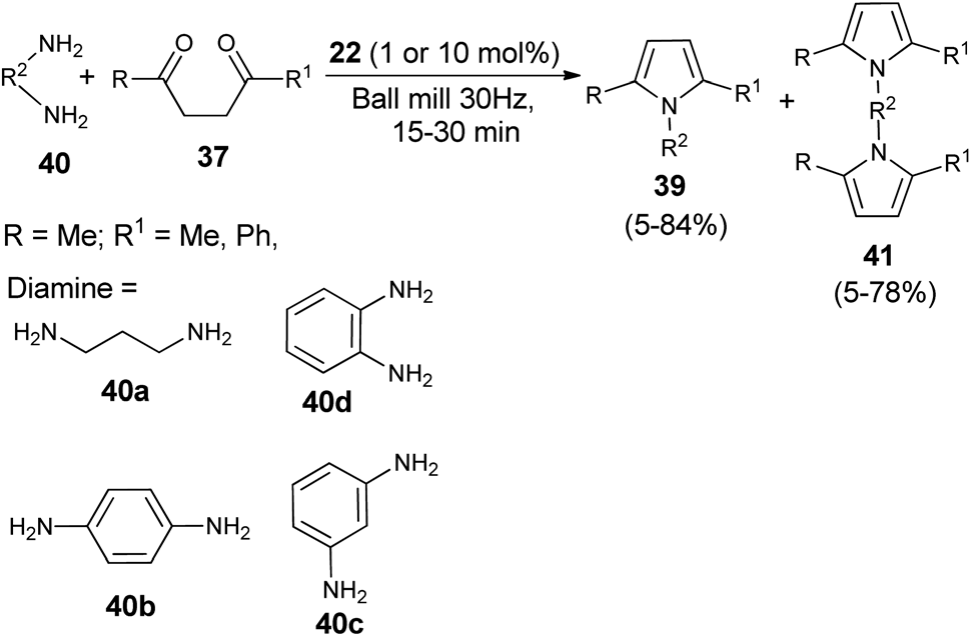
Synthesis of pyrroles by using diamines as the reactants.

A very efficient straightforward method for the synthesis of *N*-substituted pyrroles 39 under solvent-free conditions by using mechanochemical activation and biomass-derived organic acid in a very short reaction time has been developed by Akelis *et al.* (Scheme 4).^21^ The synthesis involving the reaction of diketones 37 with various aliphatic and aromatic amines 38 in the presence of citric acid 22 at 30 Hz ball-mill frequency for 15–30 minutes was found to lead to corresponding pyrroles 39 in 23–84% yield. In addition, they further extended the methodology for the desymmetrization of amines or to access bis(pyrroles) 41 by using various aromatic and aliphatic diamines 40 as the reactants under the same reaction condition. The formation of mono-pyrroles 39, *i.e.*, desymmetrization of amines and bis(pyrroles) 41 depends on the reactant diketones 37 and diamines 40 (Scheme 5).

**Scheme 6:**
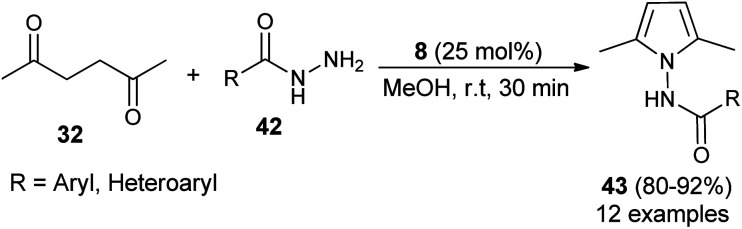
Saccharin-catalyzed synthesis of pyrroles 43.

In 2015, Bhandari and Gaonkar synthesized a series of *N*-substituted 2,5-dimethylpyrroles 43 through the two-component Paal–Knorr cyclo-condensation reaction of hexane-2,5-dione 32 with several aromatic hydrazides 42 in methanol catalyzed by 25 mol% of saccharin (8) at room temperature for 30 minutes (Scheme 6).^22^ The methodology offers several significant advantages, including non-toxicity, low cost, ecological safety, easy isolation of the product, and reusability of the catalyst that could be applicable to a wide-ranging substrate scope in good to excellent yield. All heterocyclic, as well as aromatic hydrazides, react equally well with hexane-2,5-dione 32 under the standard condition to afford the product 36.

**Scheme 7:**
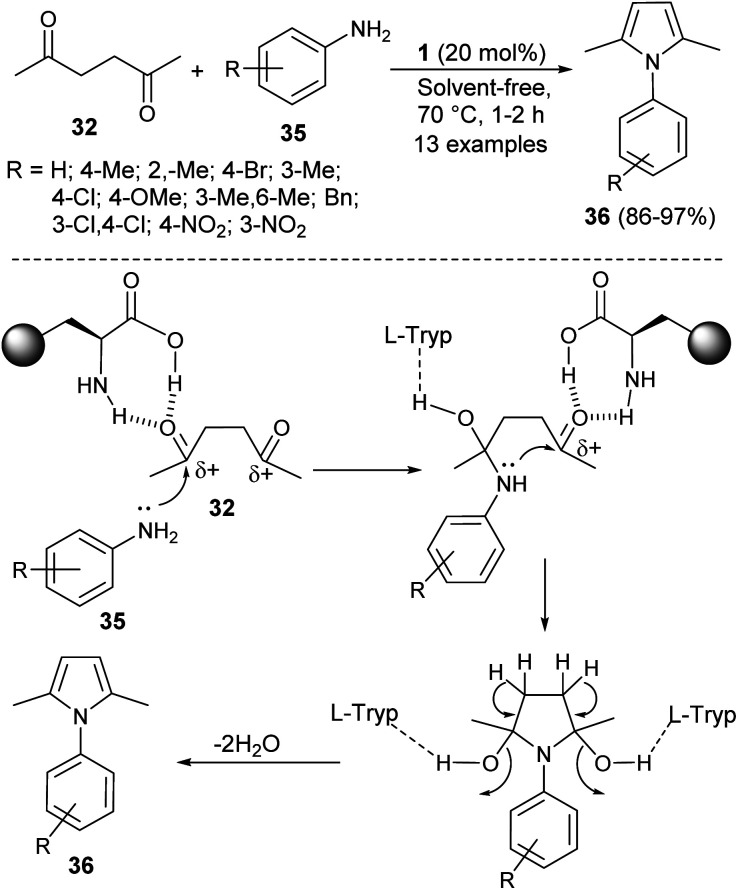
Synthesis of pyrroles *via* double-condensation reaction.

In 2016, Aghapoor *et al.*^23^ reported that the treatment of hexane-2,5-dione 32 with several aromatic amines 35 in the presence of the natural primary amino acid l-tryptophan 1 as an organocatalyst at 70 °C under solvent-free condition afforded the corresponding *N*-substituted pyrroles 36 in 86–97% yield in 1–2 hours (Scheme 7). The proposed mechanism for this transformation initiated by the double condensation of hexane-2,5-dione 32 with amines 35 under the presence of catalyst 1. The catalyst 1 activates the dicarbonyl compound by forming hydrogen bond between the carbonyl oxygen and its amino acid group, and thereby facilitating the nucleophilic attack of N-atom of aromatic amines to the carbonyl carbon. In the final stage, the subsequent removal of water molecules followed by detachment of the catalyst leads to the formation of product 36.

### 2.2 From tetrahydro-2,5-dimethoxyfurans and amines

**Scheme 8:**
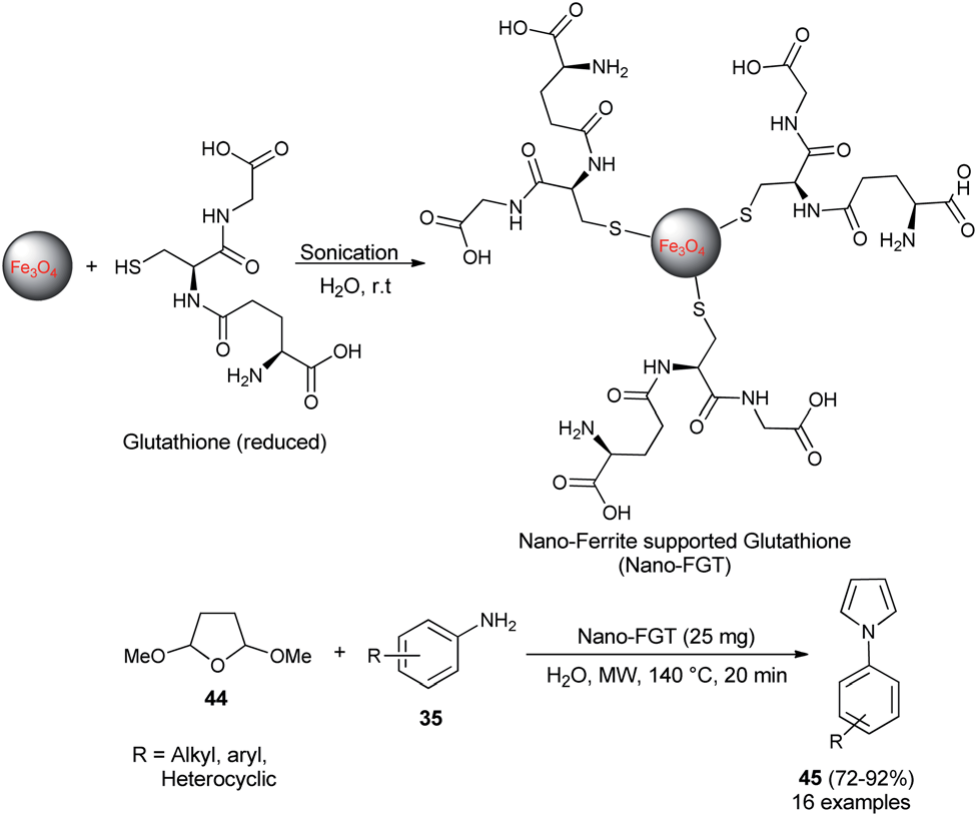
Nano-FGT catalyzed synthesis of pyrroles 45.

In 2009, Polshettiwar *et al.* reported the synthesis of a novel nanoparticle-supported organocatalyst, namely, Nano-Ferrite supported Glutathione (Nano-FGT) for the synthesis of pyrroles. The catalyst was prepared *via* the immobilization of naturally abundant tripeptide glutathione as an organocatalyst on the nano-ferrite surfaces. The catalytic activity of the prepared nanoparticle-supported organocatalyst was found to be very efficient for the two-component Paal–Knorr condensation reaction of tetrahydro-2,5-dimethoxyfuran 44 with different amines 35 in aqueous medium under microwave irradiation at 140 °C. The corresponding pyrrole 45 was obtained in 72–92% within 20 minutes (Scheme 8).^24^ Under the standard reaction condition, as mentioned, various aryl, heteroaryl, and alkyl amines worked well and a total of 16 compounds were synthesized in good to excellent yield.

**Scheme 9:**
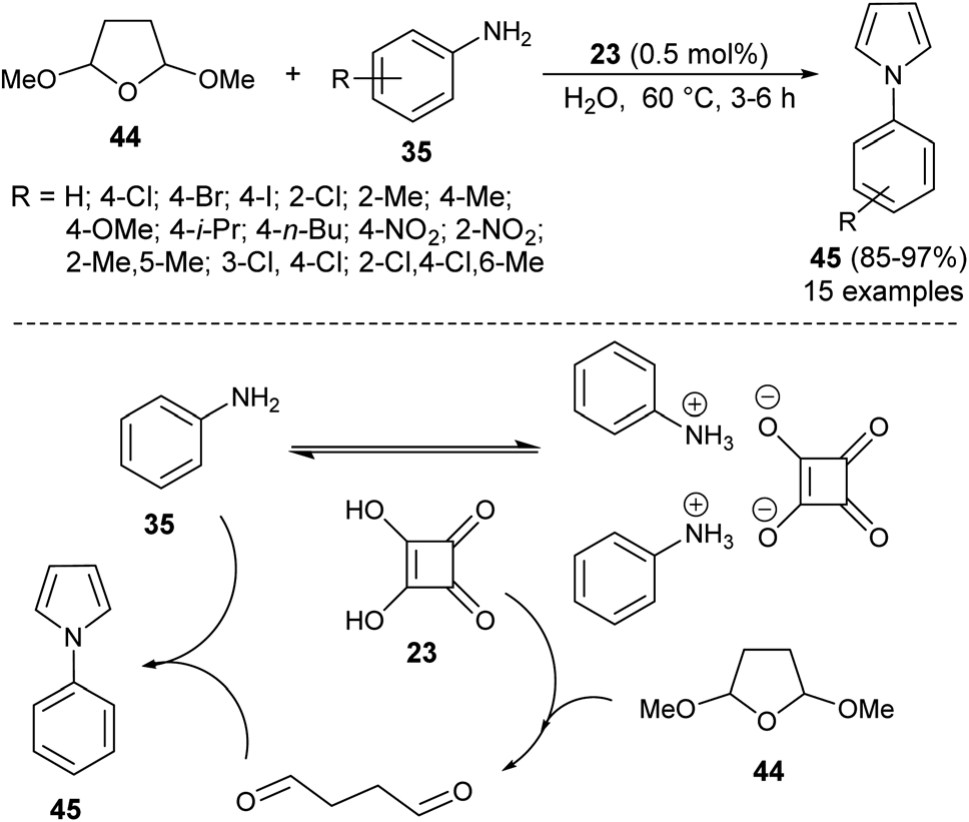
Preparation of *N*-substituted pyrroles 45 from tetrahydro-2,5-dimethoxyfuran and amines.

Another organocatalytic route for the synthesis of *N*-substituted pyrroles 45 in 85–97% yield has been accomplished *via* the treatment of tetrahydro-2,5-dimethoxyfuran 44 with several aryl amines 35 in the presence of 23 as an organocatalyst in the aqueous medium at 60 °C for 3–6 hours (Scheme 9).^19^ The suggested way for this transformation starts with the formation of anilinium squarate salt by the reversible acid–base treatment of aniline with squaric acid 23. The hydrolysis of tetrahydro-2,5-dimethoxyfuran in the presence of a catalytic amount of 23 gave the active 1,4-dicarbonyl compound, which could undergo a cyclo-condensation reaction with aniline to afford the corresponding product 45 (Scheme 9).

### 2.3 From α,β-unsaturated carbonyl compounds

**Scheme 10:**
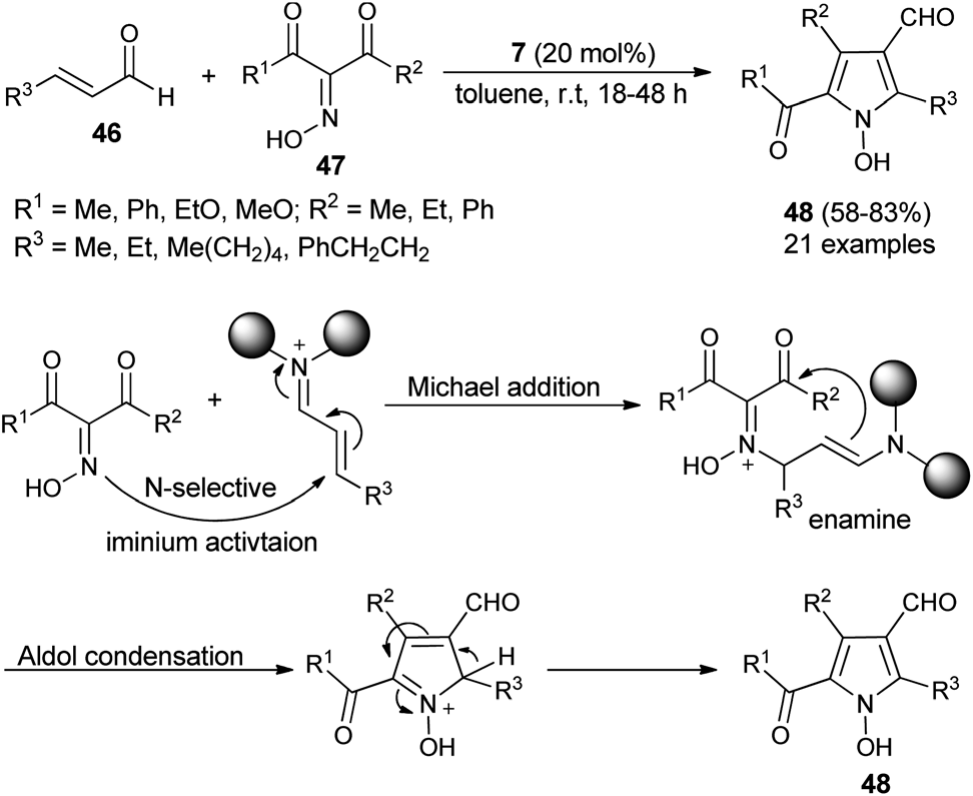
Synthesis of *N*-hydroxypyrroles 48*via* Michael addition/aldol condensation reaction.

Due to the attractive advantages, organocatalytic domino reactions have been considered as a powerful tool in organic synthesis from the last decade. In 2009, Tan and his co-workers demonstrated that the one-pot domino reaction of α,β-unsaturated aldehydes 46 and α-carbonyl oximes 47 by using 7 as an organocatalyst in toluene at room temperature for 18–48 hours afforded *N*-hydroxy pyrroles 48 in 58–83% yield (Scheme 10).^25^ The reaction has proceeded *via* the domino Michael addition/aldol condensation reaction, and oximes were utilized as *N*-selective nucleophiles for the Michael addition reaction step. The proposed mechanism involves the initial iminium activation of α,β-unsaturated aldehydes by secondary amine catalyst 7 that undergo Michael addition reaction by experiencing a nucleophilic attack from the *N*-selective nucleophile oximes. The subsequent intramolecular aldol condensation reaction and aromatization reaction afforded the final *N*-hydroxypyrroles 48 in good yield.

**Scheme 11:**
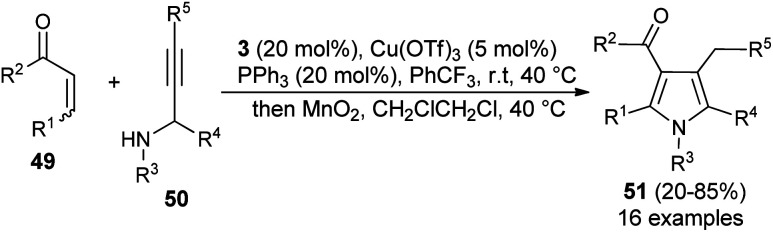
One-pot synthesis of 3-acyl pyrrole from an unsaturated ketone by cooperative catalysis.

Similar to the α,β-unsaturated aldehydes, the reactivity of unsaturated ketones was also explored for the synthesis of polysubstituted pyrroles *via* cooperative catalysis. In recent years, cooperative catalysis has drawn much more attention for the production of useful structural units by combining both metal-catalyst and organocatalyst. Treatment of unsaturated ketones 49 with *N*-substituted propargylated amines 50 by using 3 as the organocatalyst in the presence of copper salt at room temperature or 40 °C produces the polysubstituted 3-acyl pyrroles 51 in 20–85% yield (Scheme 11).^26^ The reaction has proceeded through the iminium activation of unsaturated ketones 49 by 3, followed by aza-Michael addition with substituted propargylamine that undergoes alkyne carbo-cyclization reaction, which leads to the formation of corresponding 3-acyl pyrroles 51 after the oxidation reaction.

### 2.4 Other two-component reactions

**Scheme 12:**
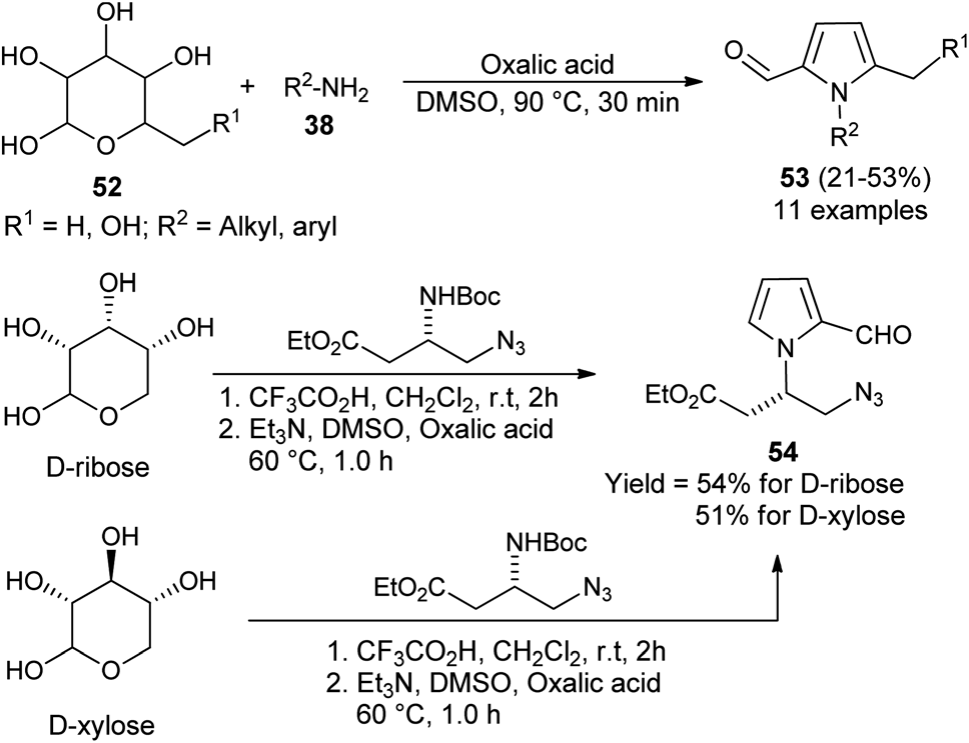
Conversion of carbohydrates into *N*-substituted pyrrole-2-carbaldehydes.

In 2015, Adhikary *et al.*^27^ reported a practical one-pot conversion procedure for the synthesis of *N*-substituted pyrrole-2-carbaldehydes 53 in 21–53% yields *via* the reaction of carbohydrates 52 with primary amines 38 in the presence of oxalic acid in DMSO at 90 °C for 30 minutes (Scheme 12). The reaction of d-ribose with amino-ester, resulting from the *N*-Boc-protected β-amino-ester, led to the formation of *N*-substituted pyrroles 54 in 54% yield. In the case of d-xylose under the same condition, the corresponding product was obtained in 51% yield.

**Scheme 13:**
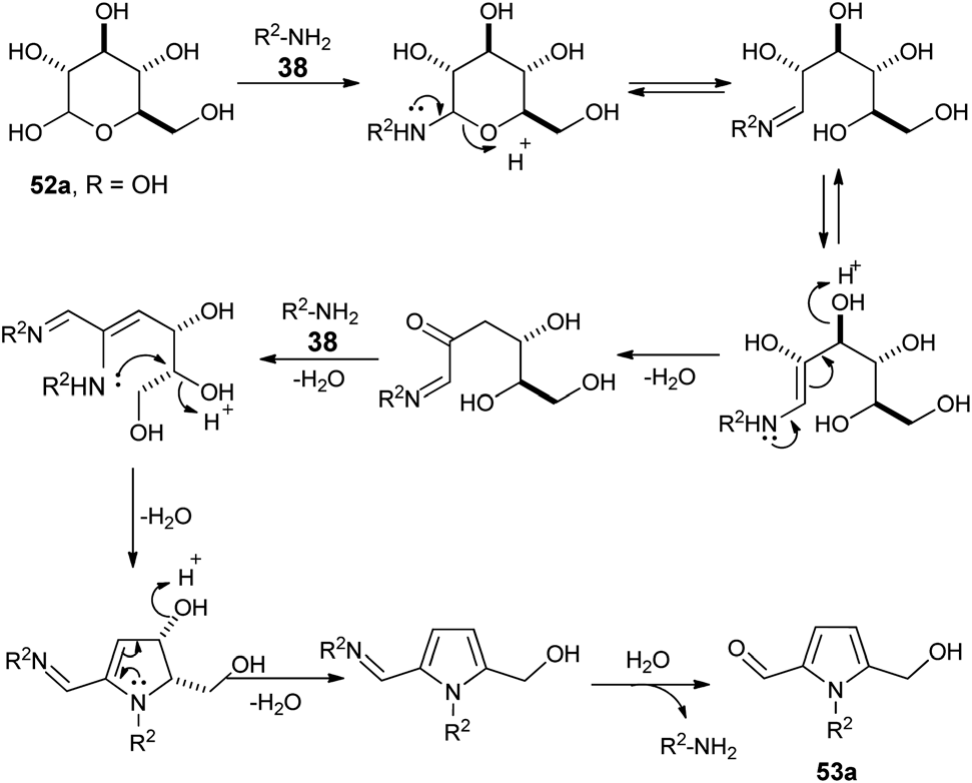
Plausible mechanism for the conversion of d-glucose 52a to pyrrole 53a.

A plausible mechanism for this practical conversion is depicted in Scheme 13. Initially, the *N*-glycosylation of amines 38 from carbohydrates 52a produces the ring-opened enamine tautomer that facilitates removal of the protonated 3-hydroxyl group to give the imine intermediate. Addition of another amine 38 to the imine intermediates, and then cyclization followed by removal of the protonated 4-hydroxyl group, and further aromatization afforded the corresponding pyrrole 53a.

**Scheme 14:**
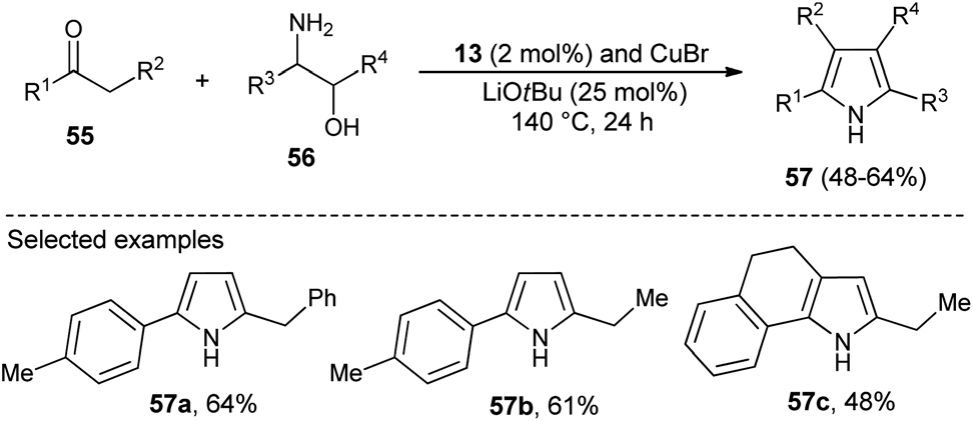
Cu–NHC catalyzed synthesis of *N*-unsubstituted pyrroles.

N-Heterocyclic carbenes (NHC) as a Lewis base organocatalyst have had a widespread impact on the organic chemistry community due to their several modes of activation. Saturated imidazolium carbene precursors 13, in combination with CuBr, catalyzed the two-component reaction of ketones 55 and β-amino alcohols 56 in the presence of base LiO*t*Bu at 140 °C for the synthesis of *N*-unsubstituted pyrroles 57 in 48–64% yield after 24 hours (Scheme 14).^28^

## 3. Synthesis of pyrroles *via* multicomponent reactions (MCRs)

### 3.1 From α,β-unsaturated compounds

**Scheme 15:**
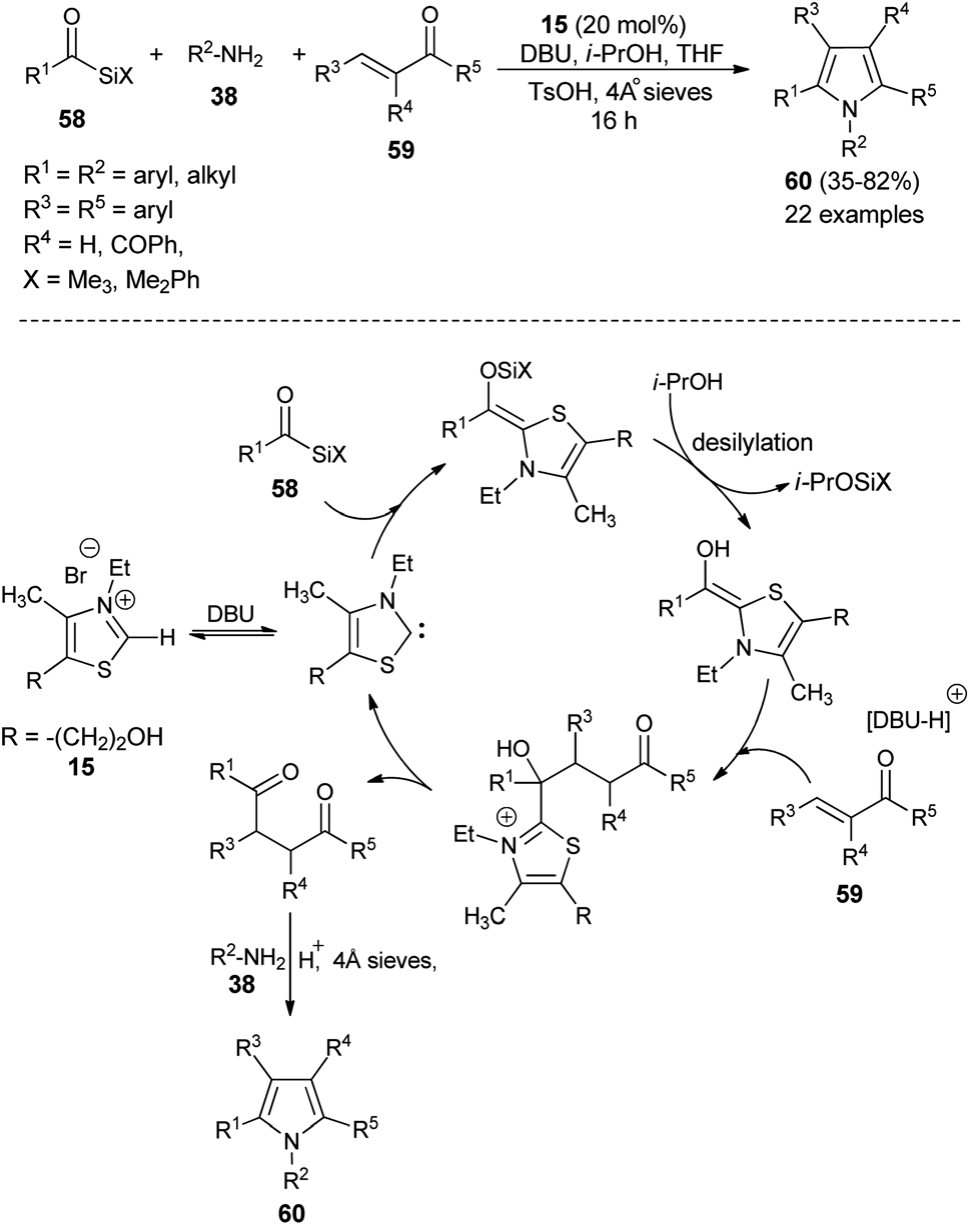
Synthesis of highly substituted pyrroles 60*via* Sila-Stetter/Paal–Knorr strategy.

In recent times, multicomponent reactions (MCRs) have increasingly gained favor in organic synthesis due to the formation of diverse molecular structures in a single step with enhanced efficiency, reduced waste, and high atom economy. In this perspective, a one-pot three-component reaction of acylsilanes 58, α,β-unsaturated carbonyl compounds 59 and amines 38 catalyzed by thiazolium salt 15 in the presence of DBU for the synthesis of highly substituted pyrroles 60*via* the Sila-Stetter/Paal–Knorr approach was developed by Ashwin and Karl in 2004 (Scheme 15).^29^ The reaction proceeded through the combination of thiazolium salt 15 with an amine base DBU that produces the N-heterocyclic carbene/zwitterionic catalyst, which facilitate the preferential acyl anion conjugate addition of acylsilanes 58 to more electrophilic α,β-unsaturated ketones 59, which leads to the formation of the 1,4-dicarbonyl compound that undergoes the Paal–Knorr condensation reaction upon treatment with amines 38, acid and dehydrating agent to produce the corresponding pyrroles 60.

**Scheme 16:**
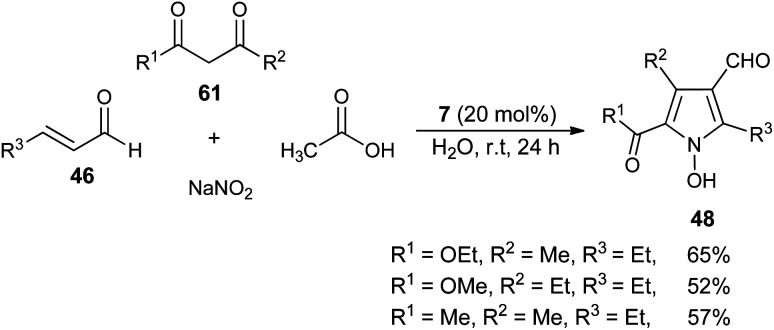
Four-component synthesis of *N*-hydroxypyrroles 48 in the presence of a secondary amine catalyst.

In 2005, a very efficient one-pot four-component reaction of α,β-unsaturated aldehydes 46, 2,4-diones 61, acetic acid, and sodium nitrite in the presence of secondary amine 7 in aqueous medium at room temperature was found to provide the green construction of polyfunctionalized *N*-hydroxypyrroles 48 after 24 hours. By using water as a green solvent, the products were isolated in moderate yield ranging from 52–65% (Scheme 16).^25^ This protocol displayed various advantages, including mild reaction condition, environmentally friendly nature, simple isolation process, low cost and the catalyst could be easily recovered.

**Scheme 17:**
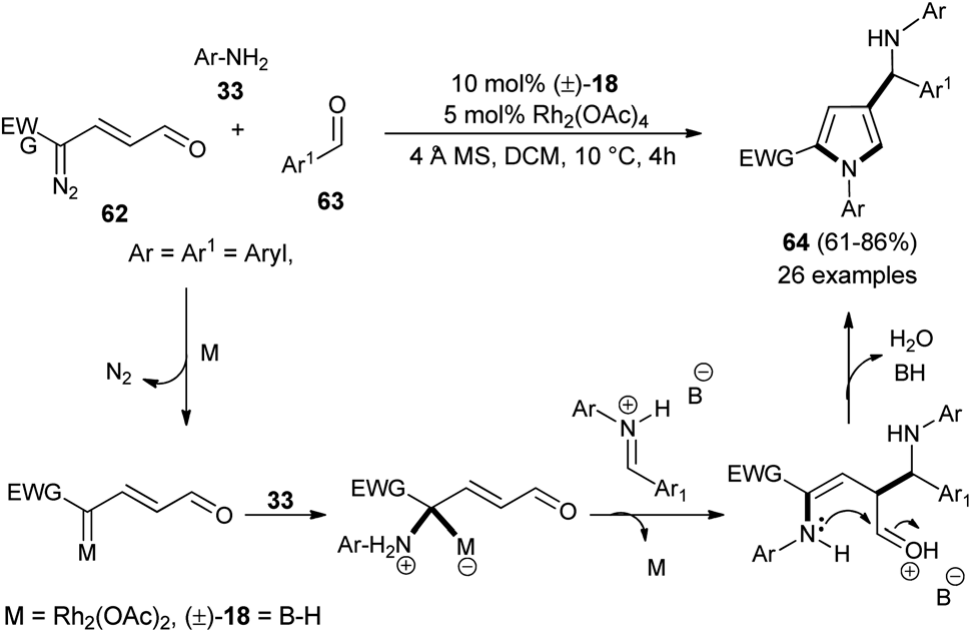
Access to pyrroles *via* ammonium ylide/Mannich reaction/cyclization cascade sequence.

Dawande *et al.* established the direct synthesis of substituted pyrroles 64 with a new stereogenic center in good yield *via* the one-pot three-component reaction of enaldiazo compounds 62, several substituted aromatic aldehydes 63, and amines 33 (Ar = Aryl, Boc) by introducing the cooperative catalyst Rh_2_(OAc)_4_ and (±)-BINOL phosphoric acid 18 in DCM at 10 °C for 4 hours (Scheme 17).^30^ It is interesting to note that due to the steric hindrance, the amine 2-(trifluoromethyl)-aniline produces the pyrrole in lower yield, whereas the amine 2,4,6-trimethylaniline did not produce the corresponding pyrrole. The methodology was found to be very efficient in the diastereoselective synthesis of the binaphthyl-based chiral pyrrole. The transient protic ammonium ylide generated from the rhodium enalcarbenoid allowing for the vinylogous nucleophilic addition to the iminium species afforded the Mannich product with a new stereogenic center that underwent [4 + 1] cyclo-condensation reaction to give the desired pyrroles 64.

**Scheme 18:**
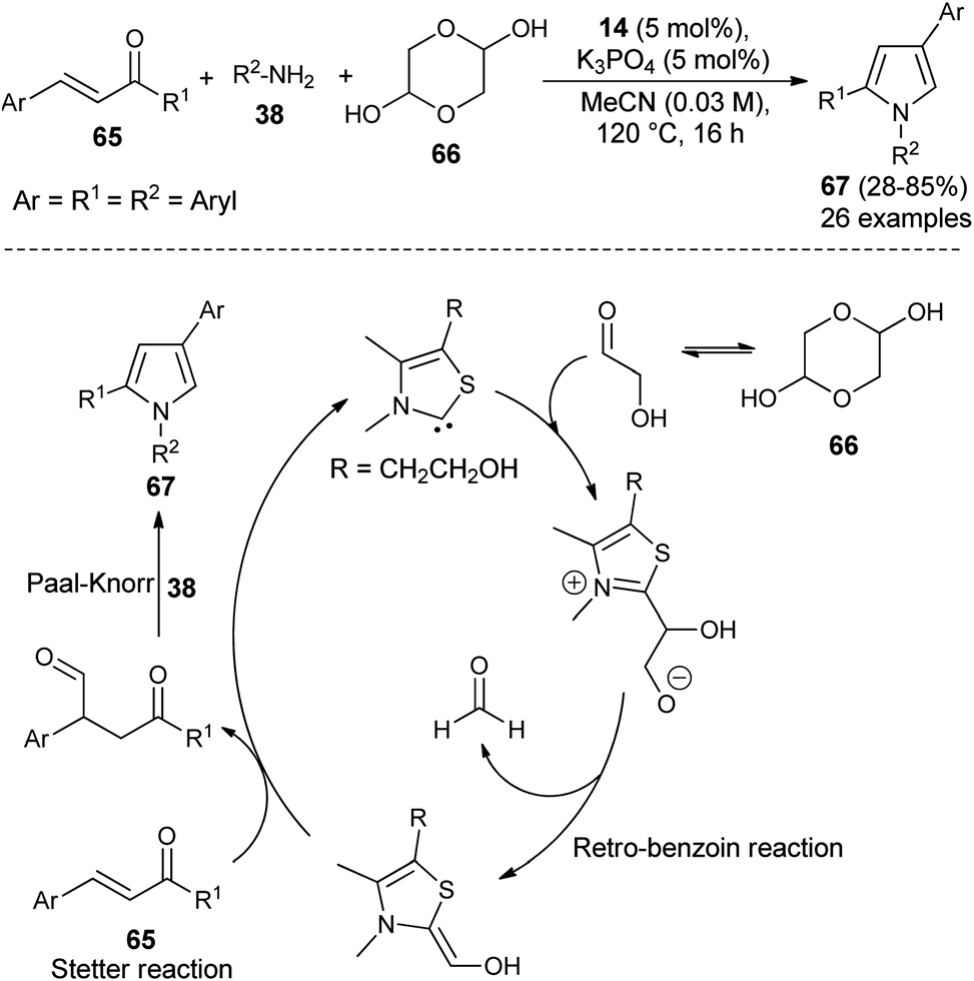
NHC-catalyzed synthesis of trisubstituted pyrroles 67.

Recently, it has been shown that the N-heterocyclic carbene catalyzed synthesis of 1,2,4-trisubstituted pyrroles could also be applicable in the synthesis of diverse structural precursors of atorvastatin. The direct one-pot three-component coupling of α,β-unsaturated ketones 65, glycolaldehyde dimer 66 as a novel C1 building block, and amines 38 using thiazolium salt 14 and K_3_PO_4_ at 120 °C in MeCN for 16 hours produced the 1,2,4-trisubstituted pyrroles 67 in 28–85% yield (Scheme 18).^31^ The mechanism proposed for this reaction sequence involves the addition of thiazol carbene (produces from the reaction of thiazolium salt 14 with K_3_PO_4_) to the carbonyl carbon of glycolaldehyde, furnishes the anionic intermediate that could undergo retro-benzoin C–C bond cleavage reactions after proton transfer, and thereby the formation of a carbon nucleophile along with formaldehyde. The conjugate addition of a carbon nucleophile with α,β-unsaturated ketones 65 as Michael acceptor *via* Stetter reaction leads to the 1,4-dicarbonyl compound that provides the corresponding pyrroles 67 after subsequent Paal–Knorr cyclo-condensation reaction with the amines 38.

### 3.2 From alkynes

**Scheme 19:**
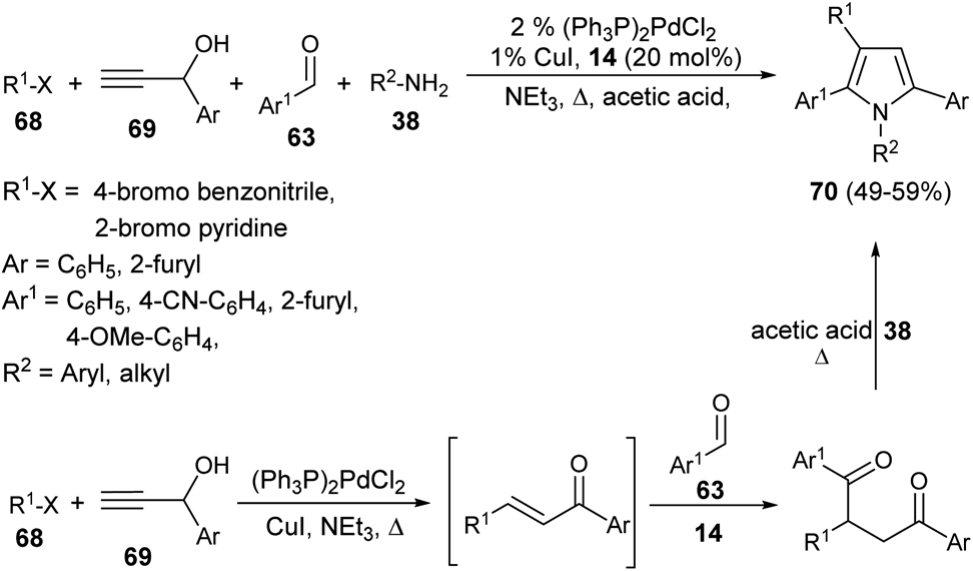
Coupling-isomerization-Stetter–Paal–Knorr strategy for the preparation of tetrasubstituted pyrroles.

In 2001, Braun *et al.* successfully synthesized a series of diverse 1,2,3,5-tetrasubstituted pyrroles 70 in 49–59% yield by exploring (hetero)aryl halides 68, such as 4-bromo benzonitrile or 2-bromo pyridine, terminal propargyl alcohols 69, aromatic aldehydes 63, and primary amines 38 as starting materials in the presence of thiazolium salt 14 (Scheme 19).^32^ This one-pot four-component method initiated by the coupling-isomerization of aryl halides 68 with propargyl alcohols 69 followed by addition of aldehydes 63*via* Stetter reaction afforded the 1,4-dicarbonyl compound, and then the subsequent Paal–Knorr reaction with amines 38 furnished the tetrasubstituted pyrroles 70.

**Scheme 20:**
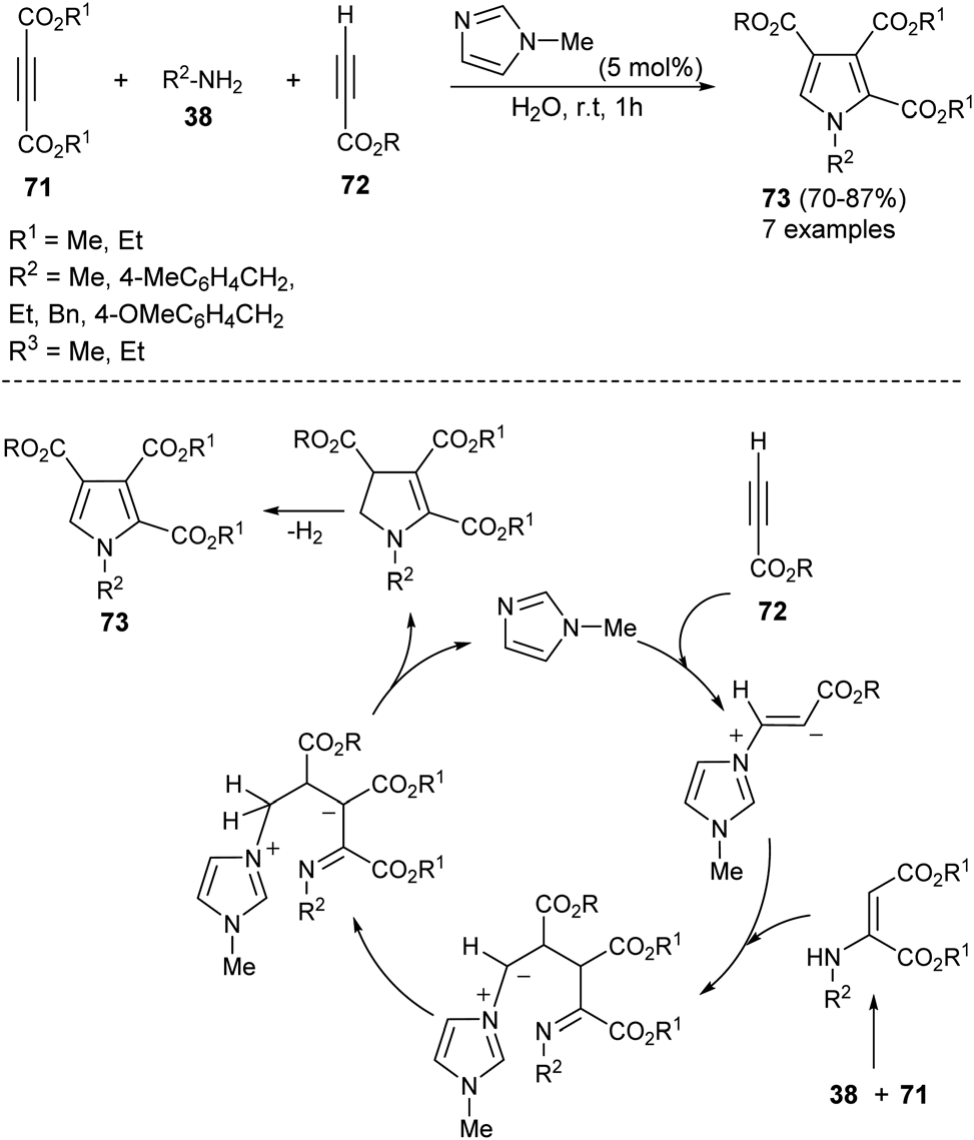
*N*-Methylimidazole catalyzed three-component synthesis of 2,3,4-trisubstituted pyrroles.

A highly efficient one-pot treatment of primary amines 38 with acetylene dicarboxylates 71 and propiolates 72 in aqueous medium by using 5 mol% *N*-methylimidazole as an organocatalyst at room temperature for 1 hour afforded the functionalized pyrroles 73 in 70–87% yield (Scheme 20).^33^ The mechanism proposed to explain this reaction begins with the addition of *N*-methylimidazole with propiolates 72, produces the zwitterionic intermediate, which undergoes addition reaction with an enamine-ester formed *in situ* from 71 and 38, followed by subsequent proton transfer and intramolecular cyclization to give the dihydropyrrole derivatives with simultaneous regeneration of the catalyst. The final elimination of hydrogen from the dihydropyrrole intermediate yielded the corresponding product 73.

### 3.3 From carbonyl compounds

**Scheme 21:**
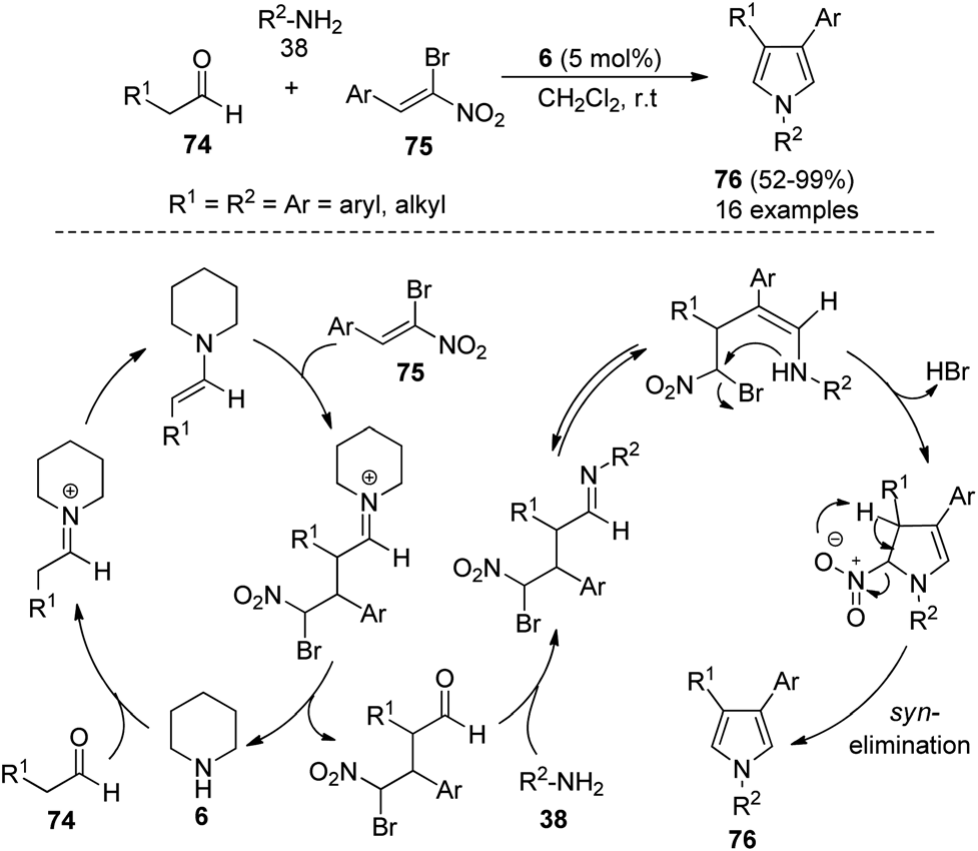
Synthesis of disubstituted pyrroles by domino MCRs.

In 2012, Martín-Santos *et al.*^34^ performed the domino three-component reaction of aldehydes 74, (*Z*)-β,β-bromo-nitroalkenes 75, and amines 38 in the presence of organocatalyst 6 in CH_2_Cl_2_ at room temperature to form the 3,4-disubstituted pyrroles 76 in good to excellent yield (Scheme 21). The mechanism involved in this reaction starts with the formation of an enamine from the addition of 6 with aldehydes 74 that undergoes Michael addition to (*Z*)-β,β-bromo-nitroalkenes 75, and produces the γ-bromo-γ-nitro-aldehyde intermediate after the hydrolysis and regeneration of organocatalyst 6. The reaction of this intermediate with amines 38, followed by tautomerization and intramolecular cyclization, and the subsequent *syn*-elimination of the nitro group afforded the final product 76.

**Scheme 22:**
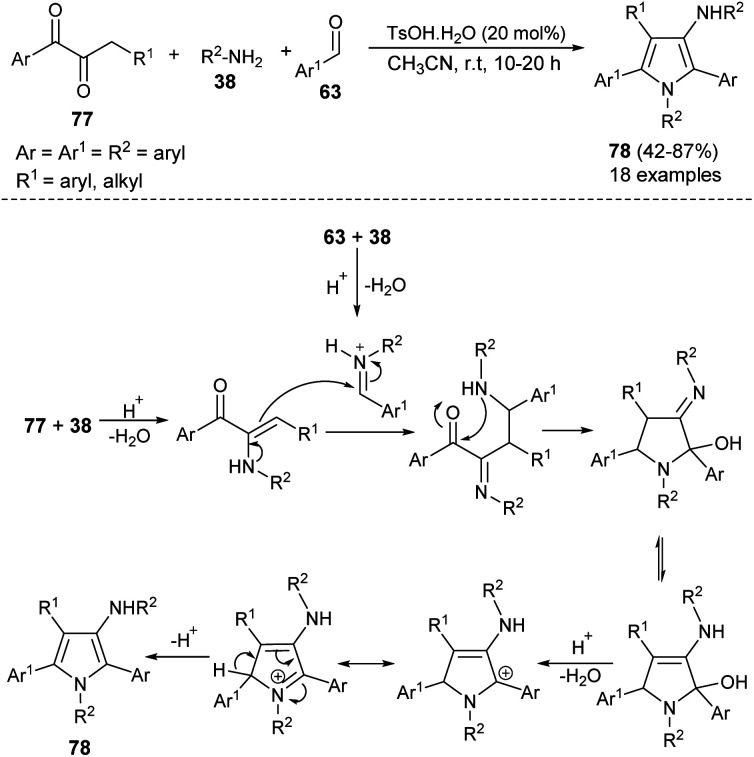
Synthesis of polysubstituted pyrroles from 1,2-diones, aldehydes, and aryl amines.

The highly efficient one-pot construction of several polysubstituted pyrroles 78 in acceptable to good yield (42–87%) has been obtained *via* the environmentally benign multicomponent reaction of 1,2-diones 77, aryl amines 38, and aldehydes 63 in the presence of 4-methylbenzenesulfonic acid monohydrate (TsOH·H_2_O) as an organocatalyst in acetonitrile at room temperature for 10–20 hours (Scheme 22).^35^ The reaction proceeded with the formation of an iminium ion from aryl amines 38 and aldehydes 63 that experiences a nucleophilic attack from the enamine intermediate, generated from aryl amines 38 and 1,2-diones 77, followed by an intramolecular cyclization and tautomerization to afford the imine form amino alcohol (Scheme 30).^25^ The loss of one molecule of water from the imine amino alcohol produces the conjugate iminium ion intermediate that yields the final product 78*via* deprotonation.

**Scheme 23:**
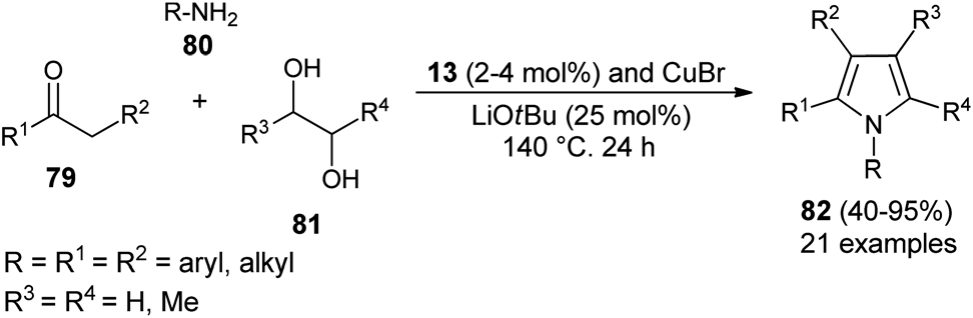
Synthesis of pyrroles by using the Cu–NHC catalyst system.

Dang *et al.*^28^ noted that the combination of CuBr with carbene precursors benzimidazolium salts 13 lead to the formation of a Cu–NHC complex as an efficient non-noble metal catalyst in the presence of a base, and was found to be a very effective catalyst in the preparation of a variety of 1,2-, 1,2,3-, variety of 1,2-, 1,2,3-, 1,2,3,5- and fully substituted pyrroles (Scheme 23). This Cu–NHC catalyzed one-pot protocol begins with the three-component reaction of different substituted ketones 79, amines 80, and diols 81 at 140 °C for 24 hours to produce the corresponding pyrroles 82 in 40–95% yields.

**Scheme 24:**
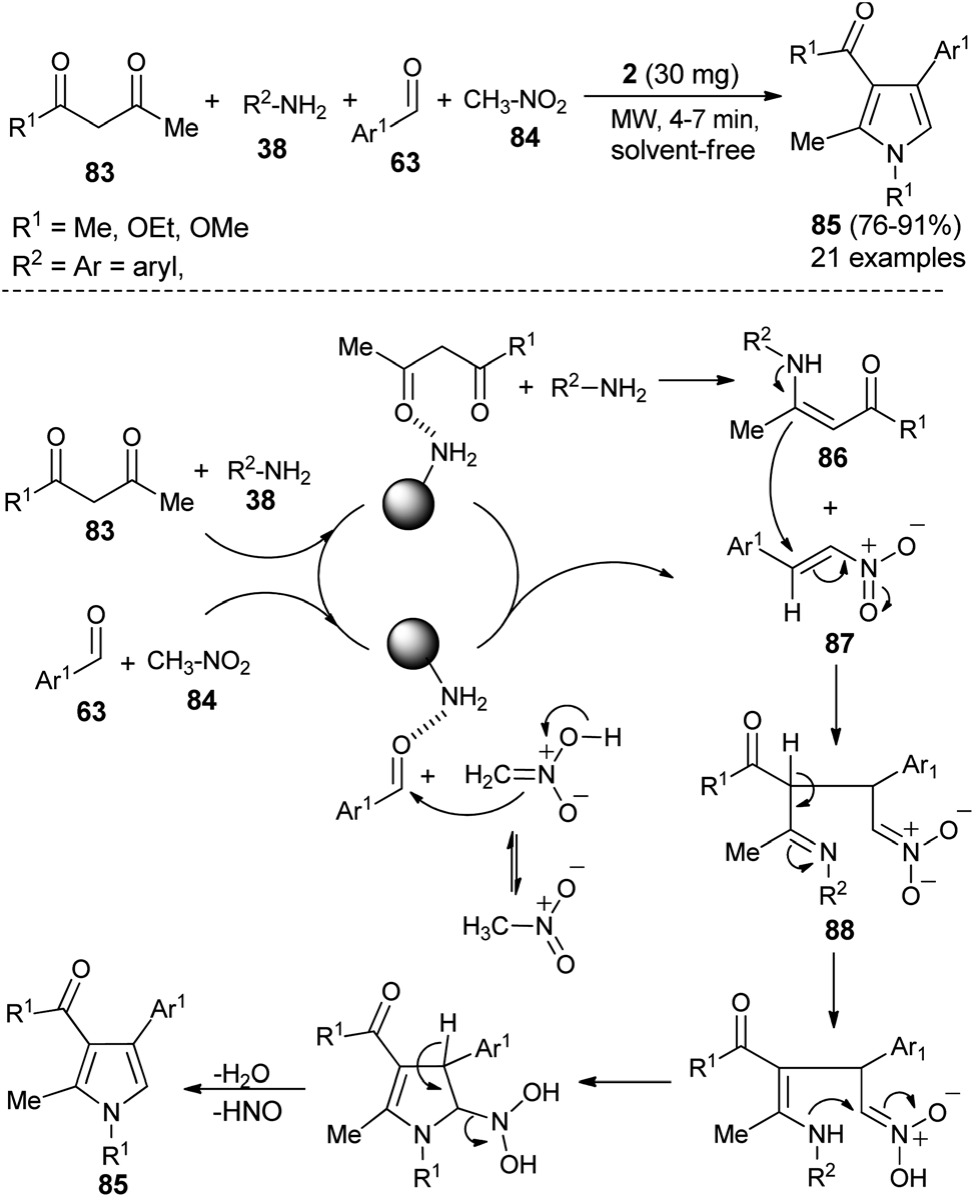
Solvent-free microwave-assisted synthesis of pyrroles.

Hassani *et al.* reported an operationally simple and eco-friendly one-pot four-component reaction of 1,3-dicarbonyl compounds 83, amines 38, aldehydes 63, and nitromethane 84 in the presence of chitosan 2 as an organocatalyst under the solvent-free and microwave-irradiation condition to afford the substituted pyrroles 85 in 76–91% yields within 4–7 hours (Scheme 24).^36^

The mechanistic pathway for this solvent-free synthesis involves the initial reaction of dicarbonyl compounds 83 with amines 38 in the presence of 2 to give the enamine intermediates 86, which react with the nitroalkenes 87 generated from the reaction of aldehydes 63 and nitromethane 84 to afford the imine 88. In both cases, the carbonyl groups were activated by the catalyst through hydrogen bonding. Through tautomerization and intermolecular cyclization, the imine 88 forms the corresponding pyrroles 85, after a subsequent loss of a water molecule and nitrosyl hydride.

**Scheme 25:**
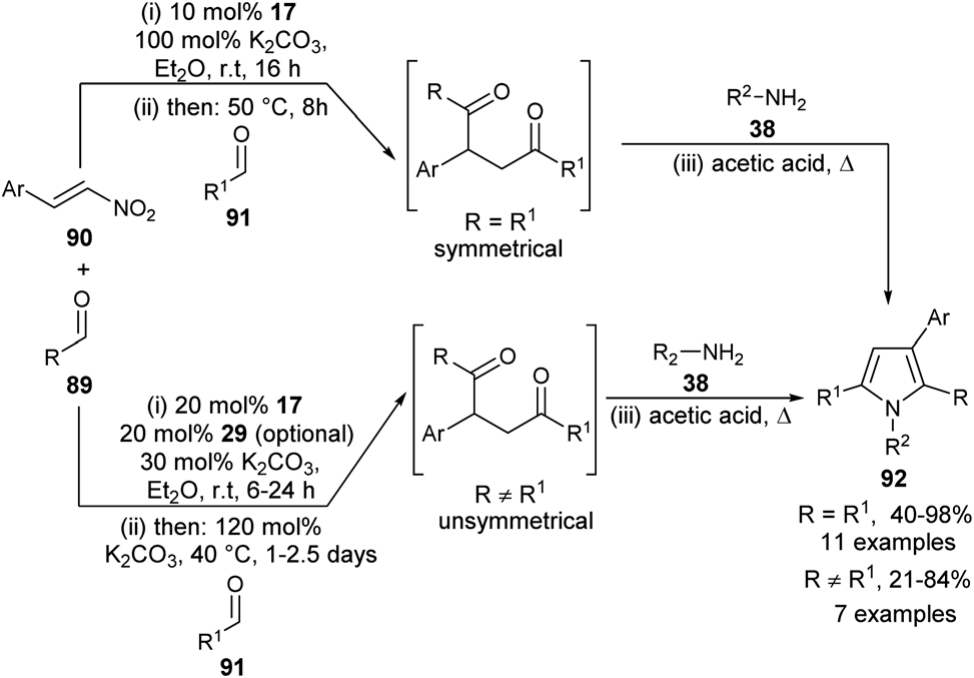
Synthesis of pyrroles *via* four-step one-pot multicomponent reaction.

A highly convergent multi-catalytic multicomponent reaction (MCR) strategy to access very constructive symmetrical and unsymmetrical 2-aryl substituted 1,4-diketone building blocks from readily available aldehydes and nitroalkenes as latent 1,2-dication synthons and their utilization for the one-pot four-step synthesis of polysubstituted pyrroles 92 by using carbene precursor 17 and K_2_CO_3_ as N-heterocyclic carbene (NHC)-catalyst has been developed (Scheme 25) by Fuchs *et al.*^37^ For the synthesis of symmetrical 1,4-diketones, the reaction of aldehydes 89 and nitroalkenes 90 was carried out in the presence of 10 mol% of NHC-precursors 17 and 100 mol% of K_2_CO_3_ in Et_2_O at room temperature for 16 hours, followed by the addition of a second aldehyde 91 at 50 °C for another 8 hours. This 1,4-diketone on treatment with amines 38 in acetic acid under heating condition afforded the corresponding symmetrical pyrroles 92 in 40–98% yield. However, for the synthesis of unsymmetrical 1,4-diketones, the amount of NHC-precursors 17 was increased to 20 mol% and the amount of base was reduced to 30 mol% in the first step, and increased to 120 mol% in the elimination step. Its final addition with amines 38 in heating acetic acid produced the unsymmetrical pyrroles 92 in 21–84% yield. In the case of reactive aldehydes, only NHC was found to be very sufficient to promote the reaction. However, in the case of aldehydes with lower reactivity, an additional thiourea derivative 29 as the H-bonding catalyst is required for the activation of 1,2-bis-electrophilic nitroalkenes.

**Scheme 26:**
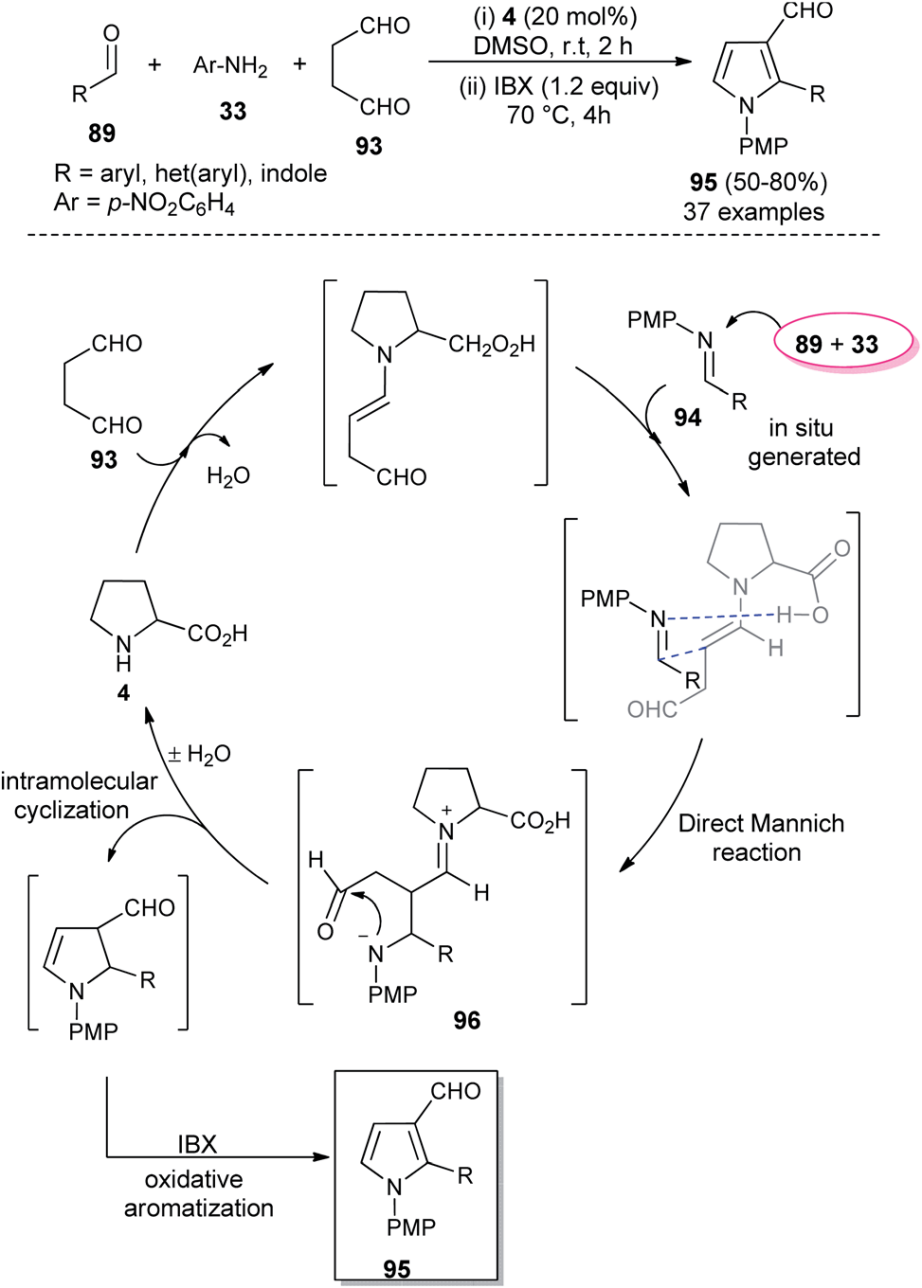
l-Proline catalyzed synthesis and mechanism of pyrroles *via* sequential multicomponent reaction.

In 2018, Singh *et al.* reported an efficient sequential multi-component strategy toward the synthesis of *N*-aryl pyrrole-3-carbaldehydes 95*via* the secondary amine 4 catalyzed reaction of aldehydes 89, arylamines 33, and succinaldehyde 93 in DMSO by using IBX as the oxidant (Scheme 26).^38^ Not only the aryl aldehydes, but also heteroaryl/indole-aldehydes worked well with this methodology, and a total of 37 compounds were synthesized in moderate to good yield. The suggested mechanism for this transformation starts with the *in situ* formation of enamine from the reaction of succinaldehyde 93 and catalyst 4, which can then react with the *N*-PMP-imines 94 generated *in situ* from aldehydes 89 and amines 33, *via* a direct Mannich reaction, resulting in the formation of Mannich product 96. The intermediate 96 then undergoes intramolecular cyclization reaction with subsequent removal of the catalyst, followed by IBX-promoted oxidative aromatization to form the final product 95.

## 4. Synthesis of pyrroles *via* multistep reactions

**Scheme 27:**
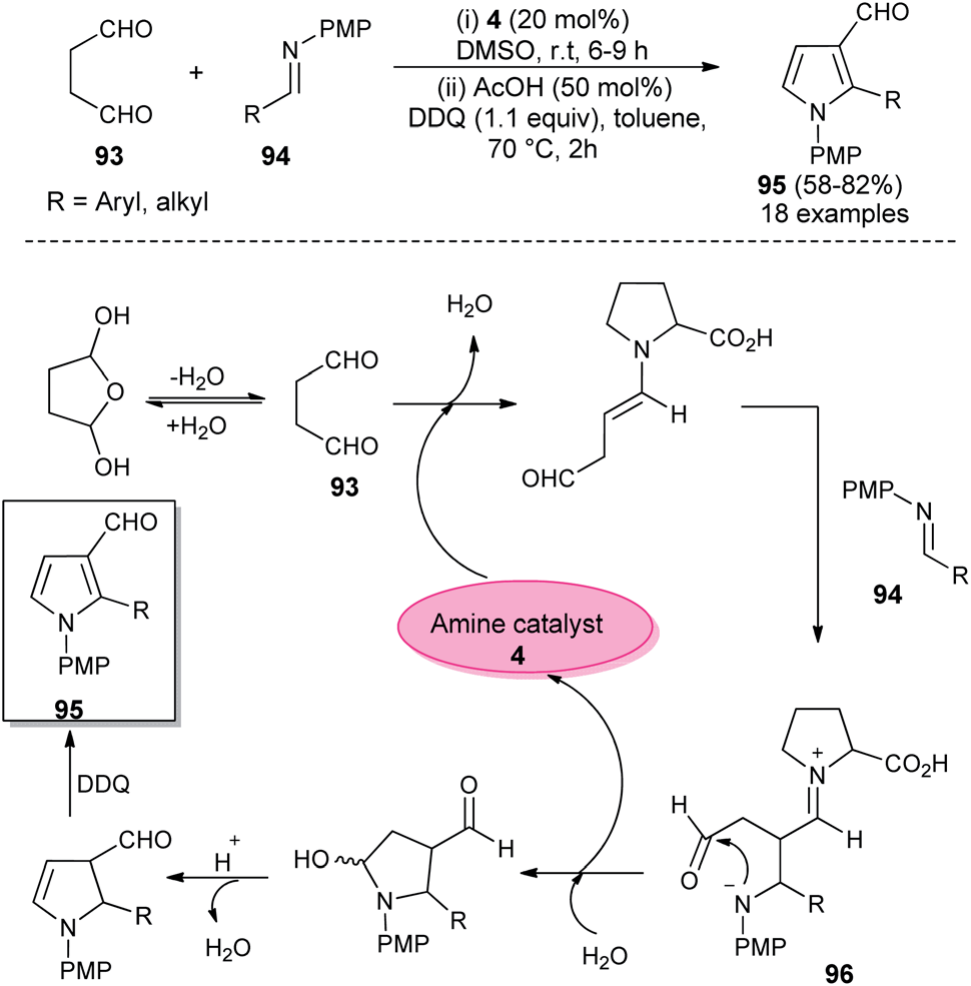
l-Proline catalyzed synthesis of trisubstituted pyrroles.

In 2012, Kumar *et al.* developed a robust two-step strategy involving the reaction of succinaldehyde 93 with *N*-PMP aldimines 94 in the presence of organocatalyst 4 in DMSO at room temperature, followed by acid-catalyzed cyclization and aromatization under the influence of DDQ in toluene at 70 °C for the synthesis of substituted pyrroles 95 in 58–82% yield (Scheme 27).^39^ This transformation was completed through the formation of an enamine intermediate 96 from the reaction of succinaldehyde 93 and amine catalyst 4, which reacts with the *N*-PMP aldimines 94*via* a direct Mannich reaction, followed by intramolecular cyclization with the simultaneous regeneration of 4, acid-catalyzed dehydration and final aromatization by DDQ with a subsequent loss of the water molecule to provide the pyrroles 95.

**Scheme 28:**
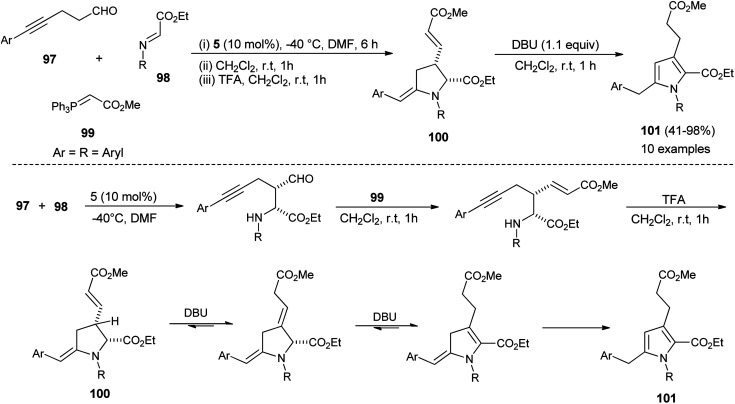
Synthesis of 2-heteroarylmethylene decorated pyrroles.

In 2013, Jean *et al.* reported the regioselective construction of 2-heteroarylmethylene decorated *N*-aryl pyrroles 101*via* two-step sequence from readily available aldehydes, imines, and phosphonium 99 in the presence of organocatalyst 5 in DMF at −40 °C. The Mannich coupling of aldehydes 97 and imines 98, followed by Wittig olefination with phosphonium 99 along with proton mediated hydroamination, leads to the rapid access of pyrrolidine 100 (Scheme 28).^40^ Isomerization of pyrrolidine 100 by simply using the amine base DBU in CH_2_Cl_2_ at room temperature afforded the substituted pyrroles 101 in 41–98% yield after 1 hour. The formation of pyrrolidine 100 and subsequent isomerization steps to 101 is shown in Scheme 28. After the formation of pyrrolidine 100, the deconjugation of the acrylate moiety followed by aromatization in the presence of the DBU base yielded the final product 101.

**Scheme 29:**
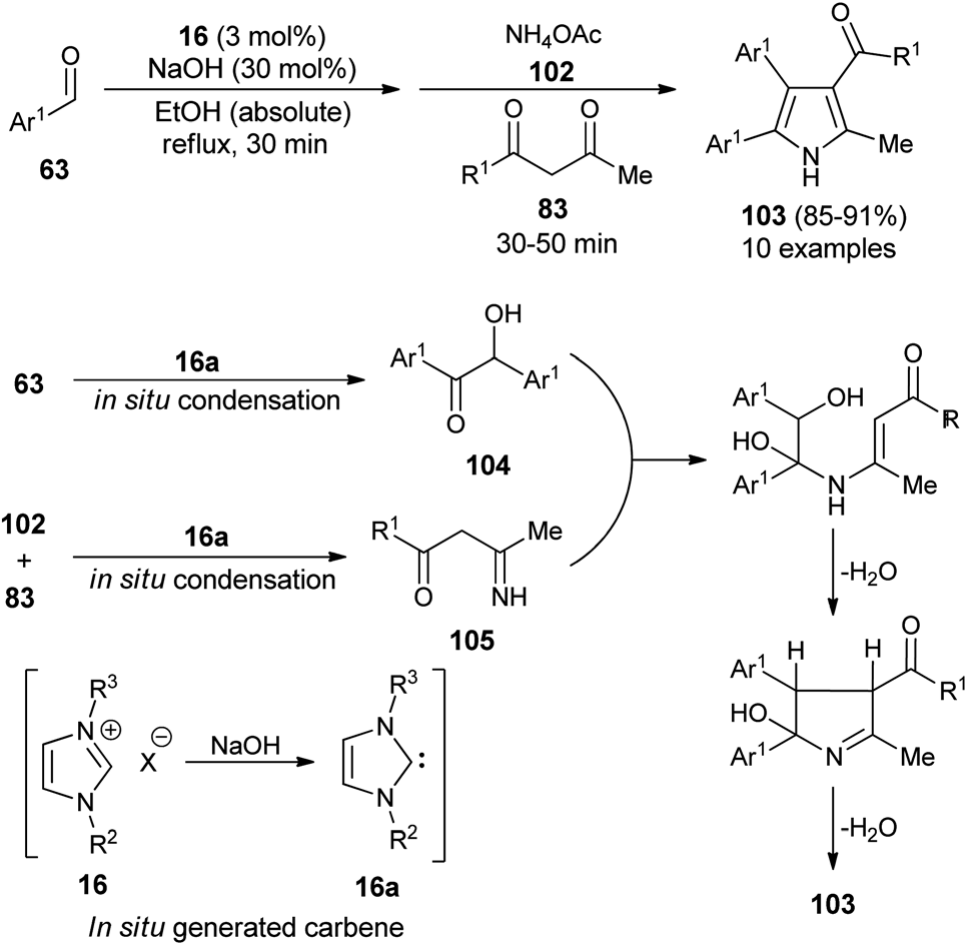
NHC-catalyzed tandem sequence to access pyrroles 103.

In 2016, Niknam *et al.* synthesized a variety of 2,3,4,5-tetrasubstituted pyrroles 103 in 85–91% yield through the one-pot multistep reaction of substituted aldehydes 63, NH_4_OAc 102, and 1,3-dicarbonyl compound 83 by using carbene precursors 16 in the presence of NaOH in absolute ethanol under reflux condition (Scheme 29).^41^ The reaction of aldehydes 63 in the presence of N-heterocyclic carbene 16a (generated *in situ* from 16 and NaOH) produced the corresponding benzoin 104*via in situ* condensation reactions after 30 minutes, which reacts with the imine 105 formed *in situ* from the NHC-mediated addition of 1,3-dicarbonyl compound 83 and NH_4_OAc 102. An intramolecular cyclization and aromatization by loss of a water molecule gave the desired product 103.

## 5. Synthesis of pyrroles *via* formal [3 + 2] cycloaddition reactions

**Scheme 30:**
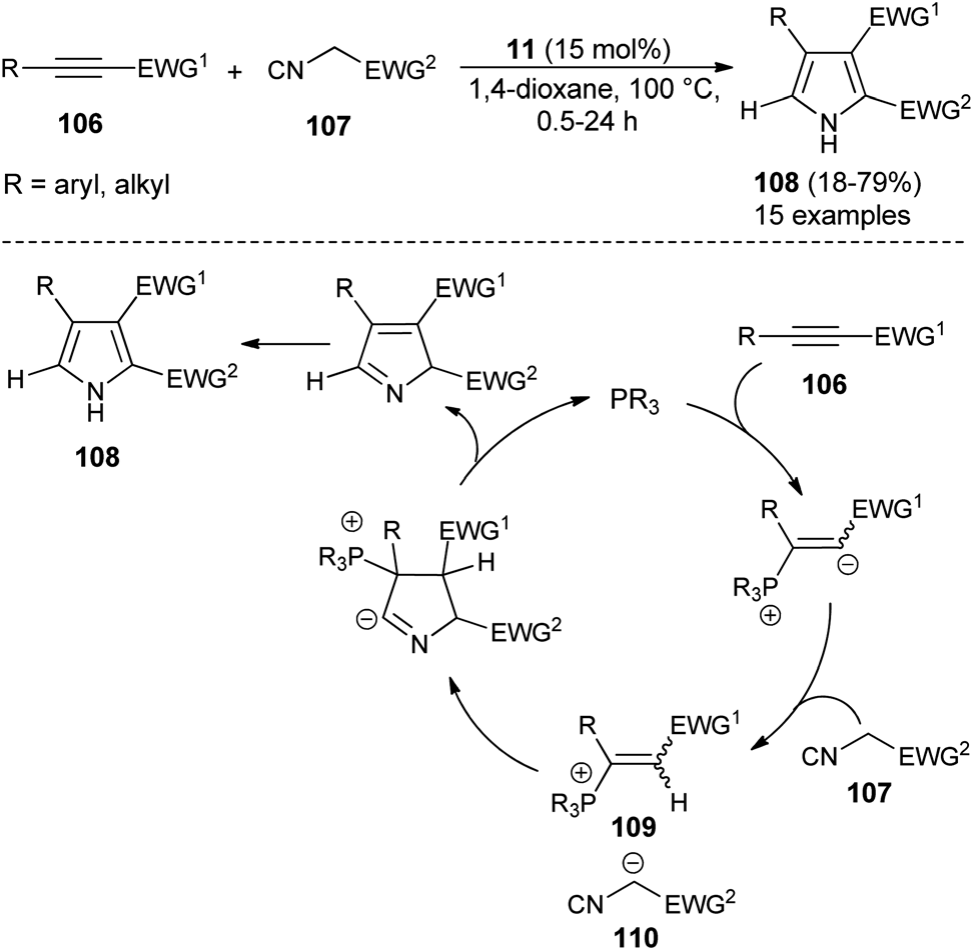
Synthesis of pyrroles from alkynes and isocyanides *via* [3 + 2] cycloaddition reaction.

For the construction of five-membered heterocyclic scaffolds, the [3 + 2] cycloaddition reaction has emerged as one of the most promising approaches, and a vast array of [3 + 2] cycloaddition reactions has been developed for the organocatalytic synthesis of pyrroles. In 2005, Kamijo and co-workers developed an organophosphine 11 catalyzed formal [3 + 2] cycloaddition reaction for the synthesis of 2,3-di-EWG-substituted pyrroles 108 from activated alkynes 106 and isocyanides 107 in dioxane at 100 °C for 0.5–24 hours in 18–79% yield (Scheme 30).^42^ The mechanism for this [3 + 2] cycloaddition reaction begins with the 1,4-addition of a nucleophilic catalyst 11 to the activated alkynes 106. It then produces the zwitterionic intermediate, which after abstraction of acidic proton in the isocyanides 107, afforded the cationic intermediate 109 and the carbanion 110 that undergoes [3 + 2] cycloaddition reaction *via* the attack of carbanion 110 to the carbon of cationic intermediate 109 bearing the EWG^1^ and thereby facilitates the generation of a new anionic center in the cationic intermediate 109 to attack the isocyanide carbon of 110, leading to the 5-membered cyclic intermediate. Its intramolecular proton relocation and removal of the catalyst followed by 1,5-hydrogen shift yields the final product 108.

**Scheme 31:**
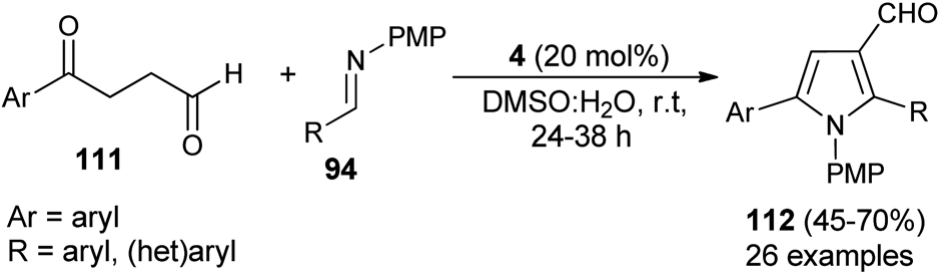
Secondary amine-catalyzed [3 + 2] annulation to pyrroles.

A straightforward route to access densely substituted 3-formyl pyrroles 112 in 45–70% yield *via* formal [3 + 2] cycloaddition reaction has been demonstrated by Kumar *et al.* in 2014. The protocol is mainly based on the one-pot cascade reaction of substituted 1,4-ketoaldehydes 111 and imines 94 in the presence of the amine catalyst 4 in aqueous DMSO at room temperature in 24–38 hours (Scheme 31).^43^ This transformation can be completed *via* the chemoselective Mannich reaction of 1,4-keto aldehydes 111 with imine 94, followed by intramolecular cyclization and aerobic oxidative aromatization.

**Scheme 32:**
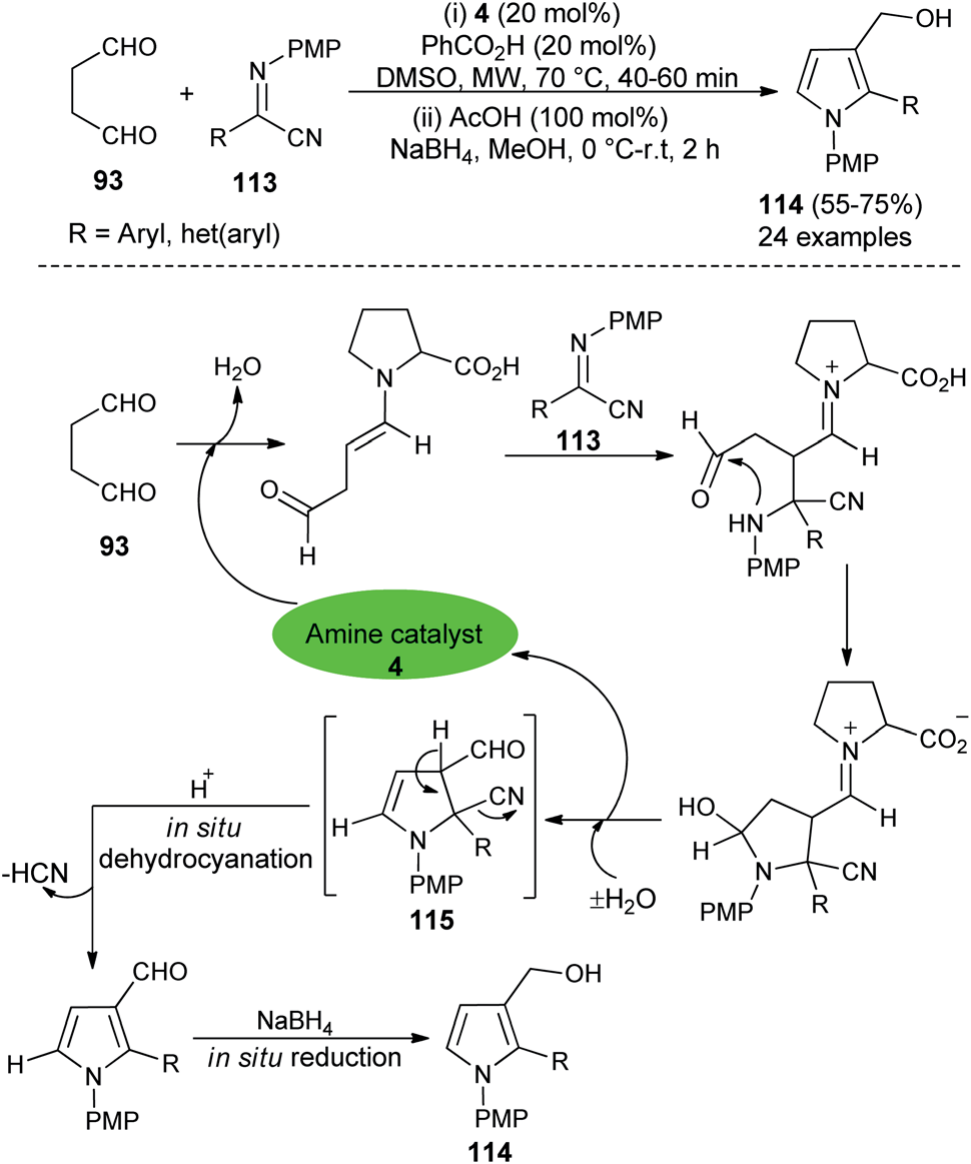
Microwave-assisted [3 + 2] annulation for the preparation of pyrroles 114.

In 2016, Mir *et al.*^44^ reported a microwave-assisted synthesis of substituted pyrrole-3-methanols 114 in good yield from the one-pot two-step reaction of succinaldehyde 93 and α-iminonitriles 113 through the amino-catalyzed [3 + 2] annulation (Scheme 32). The reaction was carried out in the presence of the secondary amine 4 in DMSO, along with PhCO_2_H as additive under microwave heating at 70 °C for 40–60 minutes, followed by the addition of cold MeOH, AcOH at 0 °C to room temperature, and then the addition of NaBH_4_ for 2 hours furnished the pyrrole-3-methanols 114 up to 75% yield. The process started with the direct Mannich reaction of an enamine intermediate generated from the reaction of 4 and 93, with iminonitriles 113, followed by intramolecular cyclization and then dehydration afforded the enamine intermediate 115. The simultaneous removal of the catalyst occurred for the next cycle. Its final *in situ* dehydrocyanation and reduction lead to the desired product 114.

**Scheme 33:**
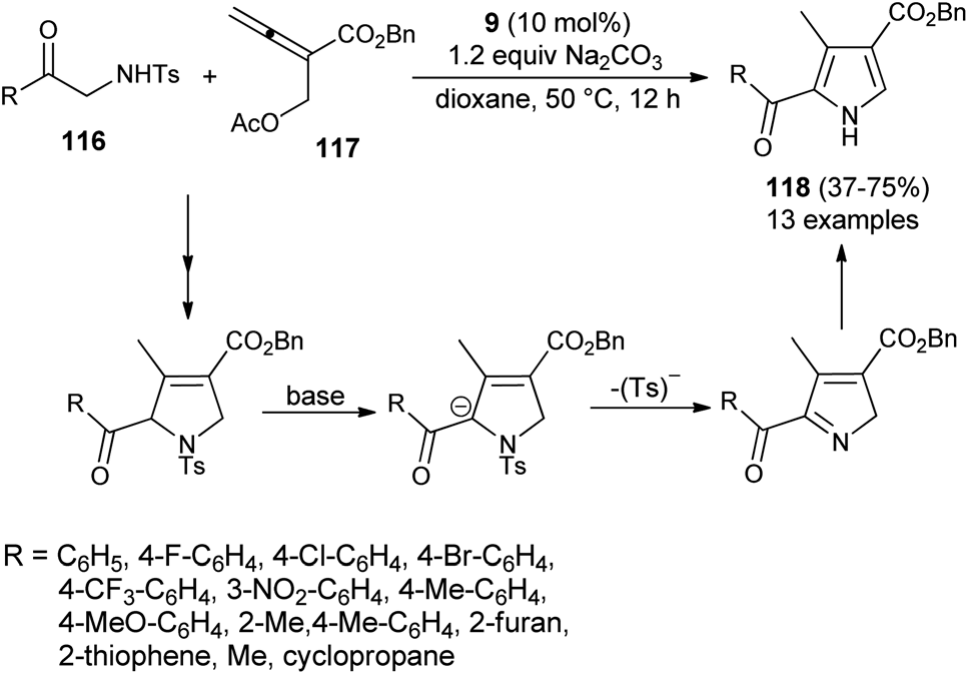
[3 + 2] Annulation/elimination/isomerization process to access substituted pyrroles 118.

In 2016, Ni *et al.* reported the [3 + 2] cycloaddition reaction to form the trisubstituted pyrroles 118 by the cascade reaction of 2-aminoketone derivatives 116 with allenoate 117 in the presence of tertiary amine 9, along with Na_2_CO_3_ in dioxane at 50 °C for 12 hours in 37–75% yield. It is pertinent to note that the 2-tosylamino ketone derivatives 116 were found to be a very efficient 1*N*,2*C*-bis-nucleophile partner for the [3 + 2] annulation with allenoate 117 (Scheme 33).^45^ However, the reaction efficiency was found to be somewhat lower due to the lower nucleophilicity of 116, and results in a moderate yield of the product, as well as incomplete reaction. The overall process involves the amino catalyzed [3 + 2] annulation of allenoate 117 and 2-aminoketone 116 that undergo 1,2-elimination of the Ts^−^ group in the presence of a base, followed by isomerization route to afford the corresponding pyrroles 118.

**Scheme 34:**
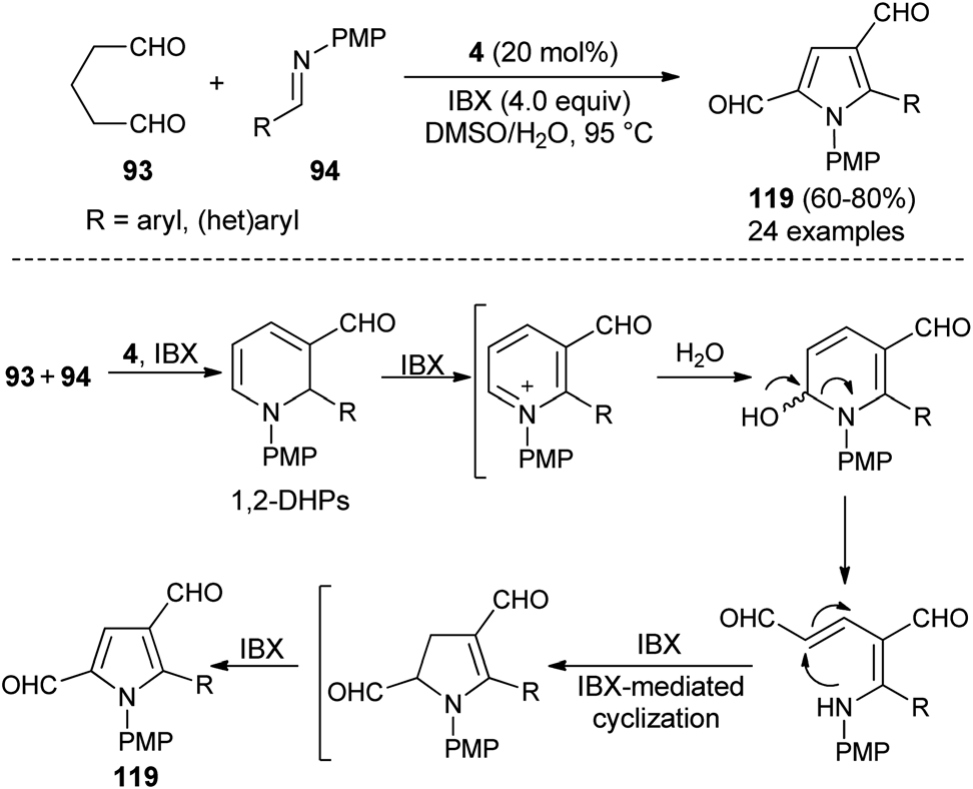
Regioselective access to pyrroles *via* pseudo-[3 + 2] annulation reaction.

Also, several substituted pyrrole-2,4-dialdehydes 119 were obtained in 60–80% yield through the one-pot reaction of glutaraldehyde 93 and imines 94 using the amine catalyst 4 in aqueous DMSO in the presence of oxidant IBX at 95 °C for 8–9 hours (Scheme 34).^46^ This was an unprecedented pseudo-[3 + 2] annulation reaction that proceeded under a metal-free condition, in which not only the substituted and unsubstituted aryl imines, but also heteroaryl imines were well tolerated in the pyrrole synthesis. The mechanism involves the direct Mannich reaction of glutaraldehyde 93 with imines 94, and then cyclization in presence of organocatalyst 4, followed by regioselective oxidation under the influence of oxidant IBX to produce 1,2-dihydropyridines (DHPs). The oxidation, ring-opening, and IBX-promoted intramolecular cyclization afforded the dihydropyrrole intermediate that undergoes final oxidative aromatization to give the pyrroles 119.

**Scheme 35:**
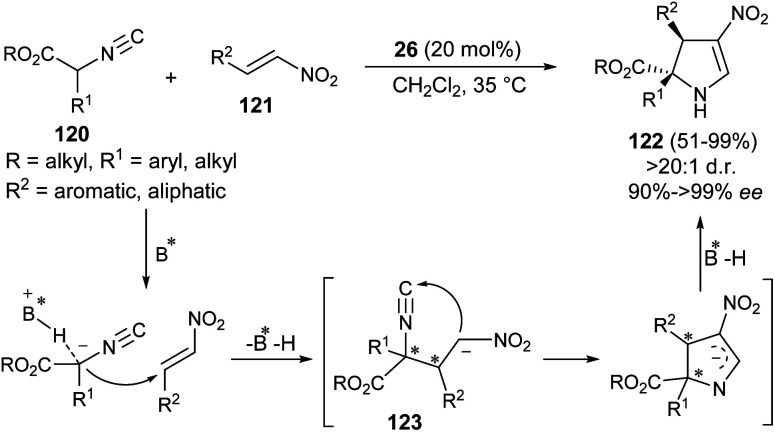
Organocatalytic formal [3 + 2] cycloaddition reaction for the asymmetric construction of dihydropyrroles 122.

Guo *et al.* developed the first organocatalytic asymmetric construction of optically active 2,3-dihydropyrroles by means of the formal [3 + 2] cycloaddition reaction. Treatment of several isocyanoesters 120 and nitroalkenes 121 in the presence of cinchona alkaloid 26 in CH_2_Cl_2_ at 35 °C was found to lead to 2,3-dihydropyrroles 122 in 51–99% yield (Scheme 35).^47^ Not only the aromatic ring bearing various electron-donating and electron-withdrawing substituents, but also aliphatic nitroalkenes were well tolerated, and resulted in the formation of the products with high diastereoselectivities of up to >20 : 1 with 91 to >99% ee. The process was initiated with the activation of the acidic α-carbon atom of isocyanoesters 120 by the catalyst 26 to undergo enantioselective Michael addition with nitroalkenes 121, thereby providing the intermediate 123. The subsequent intramolecular cyclization of 123 followed by protonation afforded the final product 122.

## 6. Synthesis of axially chiral pyrroles

**Scheme 36:**
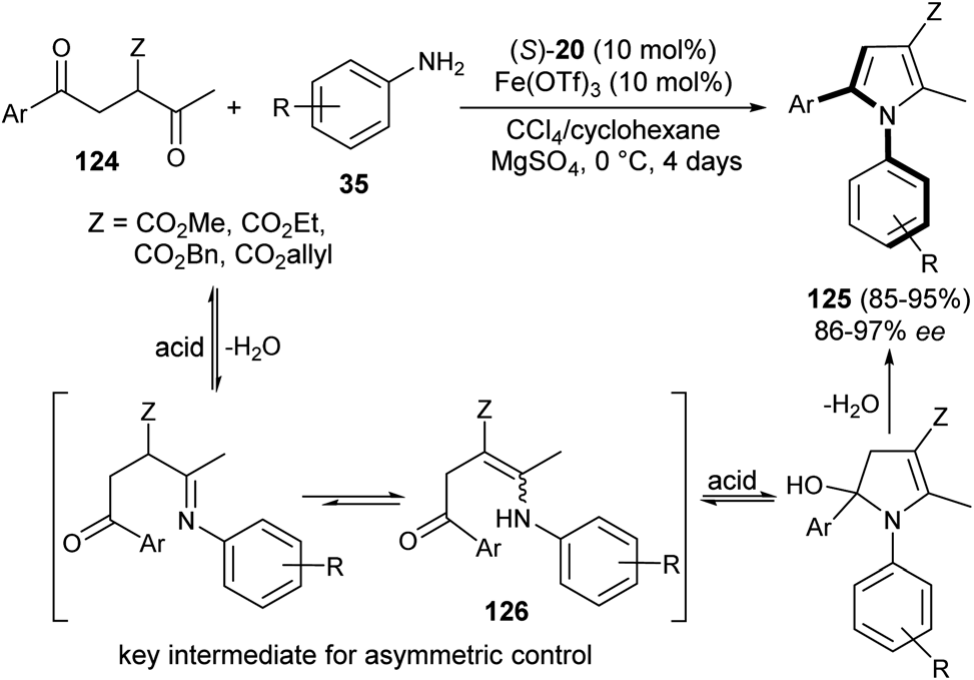
Atroposelective construction of pyrroles 125*via* Paal–Knorr reaction strategy.

The existence of organocatalysis has led to a chiral revolution in the field of asymmetric synthesis.^48–51^ The growth of asymmetric organocatalysis in the synthesis of pyrroles has drawn much more attention due to the inexpensive, metal-free, and non-toxic reaction conditions. In this context, Zhang *et al.* reported a very efficient strategy for the synthesis of axially chiral aryl pyrroles 125 with high enantioselectivity by introducing a chiral phosphoric acid (*S*)-20 as the organocatalyst and Fe(OTf)_3_ as the Lewis acid. The combination of two acid systems enhances the enantioselectivity of the corresponding aryl pyrroles 125 (Scheme 36).^52^ The reaction of various 1,4-diketones 124 with aromatic amines 35 using the combined acid catalyst system in CCl_4_ and cyclohexane at 0 °C afforded the chiral aryl pyrroles 125 in 85–95% yield with 86–97% ee after 4 days. This highly atroposelective transformation of the chiral aryl pyrroles initially involves the formation of the key enamine intermediate 126 from 1,4-diketones 124 and amine 35, which then undergo acid-catalyzed dehydrative cyclization to produce the desired product 125.

**Scheme 37:**
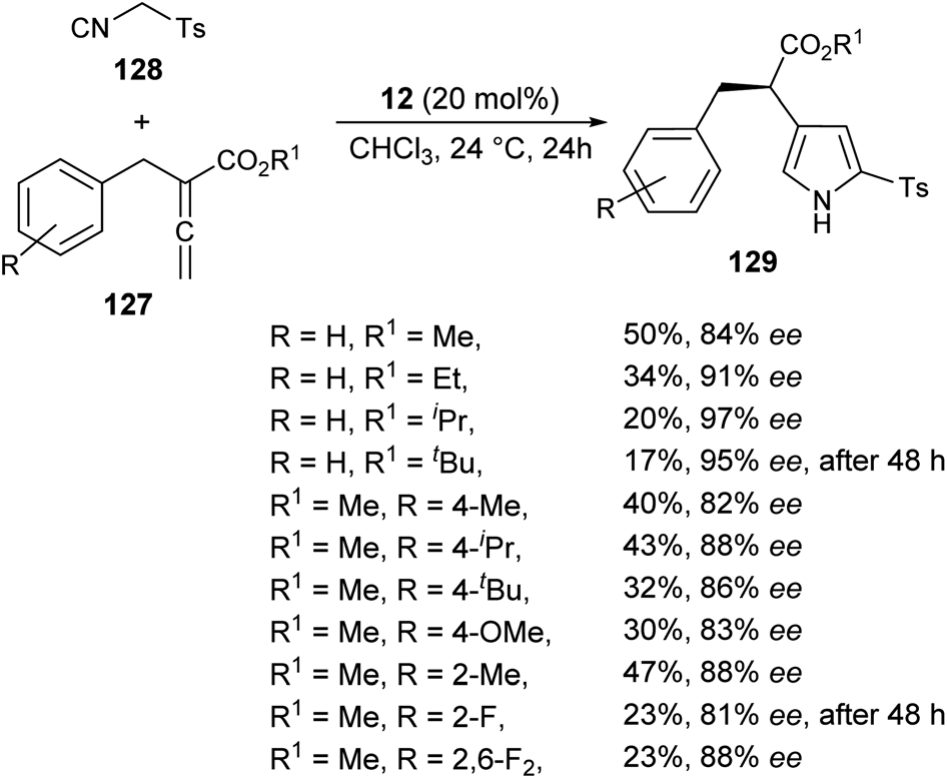
Enantioselective synthesis of substituted pyrroles 129*via* [3 + 2] cycloaddition sequence.

The chiral phosphine-catalyzed synthesis of enantioenriched 1*H*-pyrroles *via* formal [3 + 2] cycloaddition reaction has been reported by Zhao and co-workers in 2018. Treatment of allenoates 127 and activated isocyanides 128 in the presence of 12 in CHCl_3_ at 24 °C for 24 hours afforded the enantioenriched pyrroles 129 in 17–50% yield with 81–97% ee (Scheme 37).^53^ Several electron-withdrawing and electron-donating substituents on the benzyl ring of allenoate affected the yield and enantioselectivity of the corresponding products. When electron-withdrawing groups were substituted at the *ortho* or 2,6-position, the yield of the product was found to be very low with good enantioselectivity. Whereas, for the electron-donating groups, substitution at the *ortho* position furnished moderate yield with good ee. Also, the electron-donating groups at the *para* position of the benzyl ring were well tolerated by this method.

**Scheme 38:**
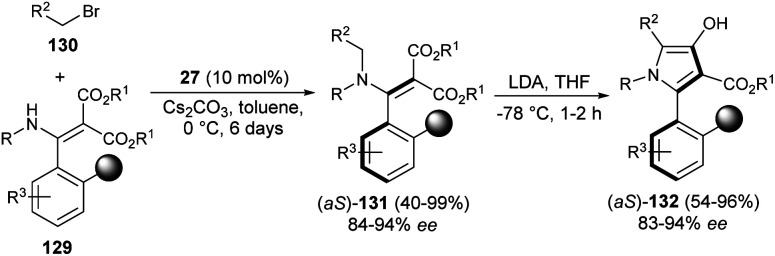
Synthesis of axially chiral 2-aryl pyrroles *via* chirality transfer approaches.

In 2019, the synthesis of axially chiral 2-aryl pyrroles 132 from enantioenriched atropisomeric alkenes *via* direct chirality transfer strategy was developed by Wang *et al.*^54^ The atropisomeric alkenes 131 were produced through the asymmetric reaction of substituted enamines 129 with *N*-alkylating reagents 130 in the presence of the cinchonine-derived organocatalyst 27 and Cs_2_CO_3_ in toluene at 0 °C for 6 days. Treatment of 131 in the presence of the strong base lithium diisopropylamide (LDA) in THF at −78 °C for 1–2 hours afforded the corresponding axially chiral 2-aryl pyrrole scaffolds 132 in 54–96% yield with 83–94% ee (Scheme 38).

**Scheme 39:**
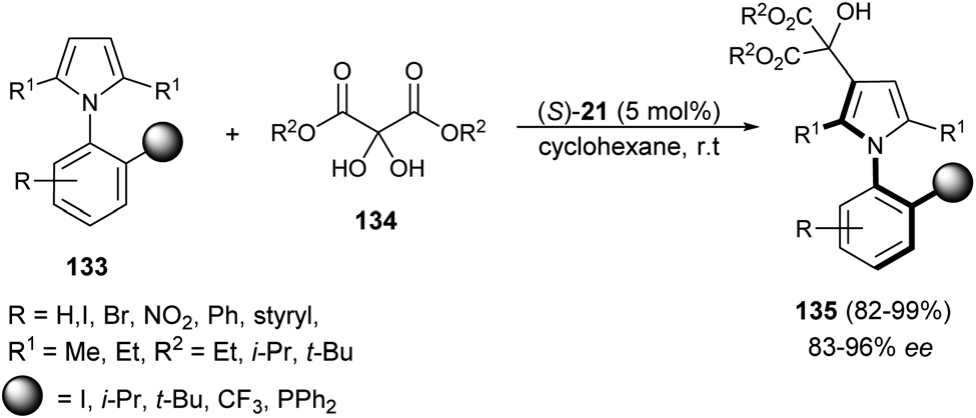
Synthesis of axially chiral aryl pyrroles 135*via* desymmetrization/kinetic resolution strategy.

Zhang *et al.*^55^ reported an atroposelective synthesis of axially chiral aryl pyrroles 135 from 1*H*-pyrrole 133 and diethyl ketomalonates 134 in the presence of chiral phosphoric acid (*S*)-21 in cyclohexane at room temperature *via* desymmetrization/kinetic resolution strategy. Products were formed in very high yield (82–99%) with 83–96% ee (Scheme 39).

**Scheme 40:**
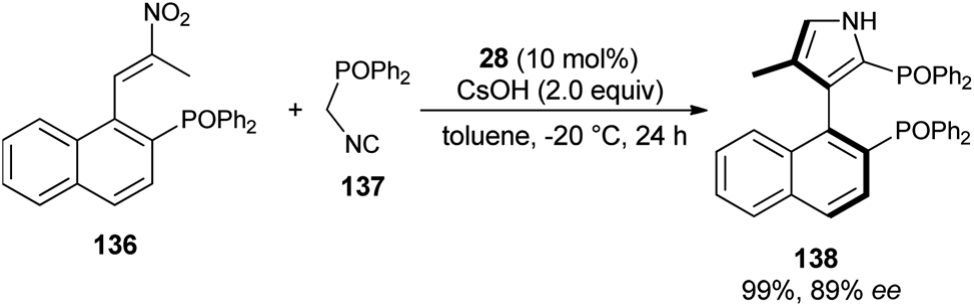
PTC-catalyzed asymmetric construction of chiral bisphosphine 138 bearing a 3-pyrrole unit.

The reaction of nitroolefin of type 136 with α-isocyanomethyldiphenylphosphine oxide 137 in the presence of the cinchona-derived phase transfer catalyst 28 and CsOH in toluene at −20 °C after 24 hours afforded the corresponding axially chiral pyrrole 138 in 99% yield with high enantioselectivity (Scheme 40).^56^ However, when the same reaction was carried out in a Ag_2_O/quinine-derived aminophosphine ligand catalytic system, the corresponding product 138 was formed in 75% yield with 21% ee.

**Scheme 41:**
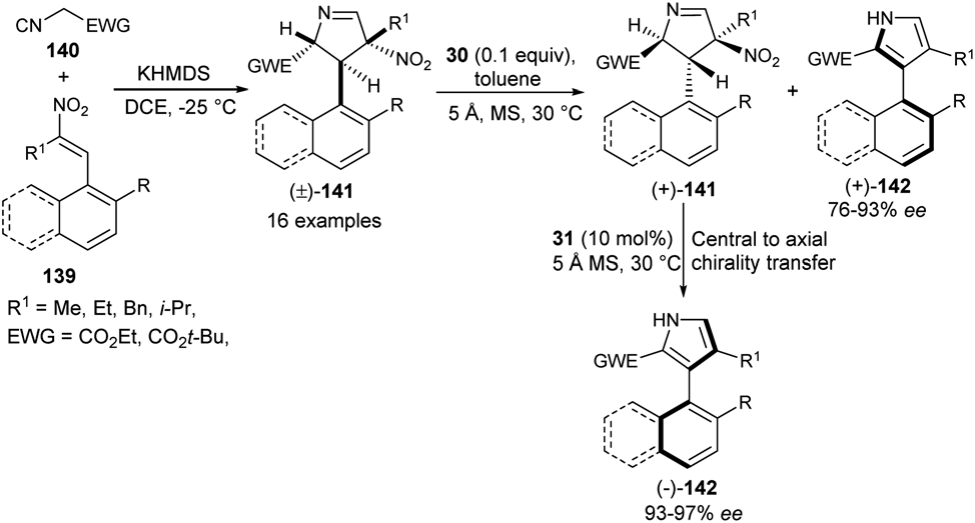
Kinetic resolution of 141*via* enantioselective aromatization sequence to access 3-aryl pyrroles 142.

In 2019, Zheng *et al.*^57^ also noted that the enantioenriched 3-aryl pyrroles would be obtained from the kinetic resolution of the racemic intermediate of the Barton–Zard reaction *via* the enantioselective aromatization reaction. The process starts with the base potassium hexamethyldisilazide (KHMDS)-catalyzed diastereoselective reaction of nitroolefins 139 with α-isocyano substrates 140 bearing an electron-withdrawing group to produce the Barton–Zard intermediate 3,4-dihydro-2*H*-pyrroles 141 as a racemic product (Scheme 41). This diastereomerically pure (±)-3,4-dihydro-2*H*-pyrroles [(±)-141] on treatment with quinine-derived thiourea 30 and 5 Å MS (molecular sieves) in toluene at 30 °C underwent enantioselective aromatization, thereby providing (+)-3-aryl pyrroles [(+)-142] in good yield with 76–93% ee, and recovered (+)-3,4-dihydro-2*H*-pyrroles [(+)-141] in 50–98% ee. The subsequent aromatization of the resolved (+)-141 in the presence of another quinidine-derived catalyst 31 in toluene at 30 °C furnishes the (−)-3-aryl pyrroles [(−)-142] in 93–100% yield with excellent central-to-axial chirality transfer.

## 7. Other strategies for the synthesis of pyrroles

**Scheme 42:**
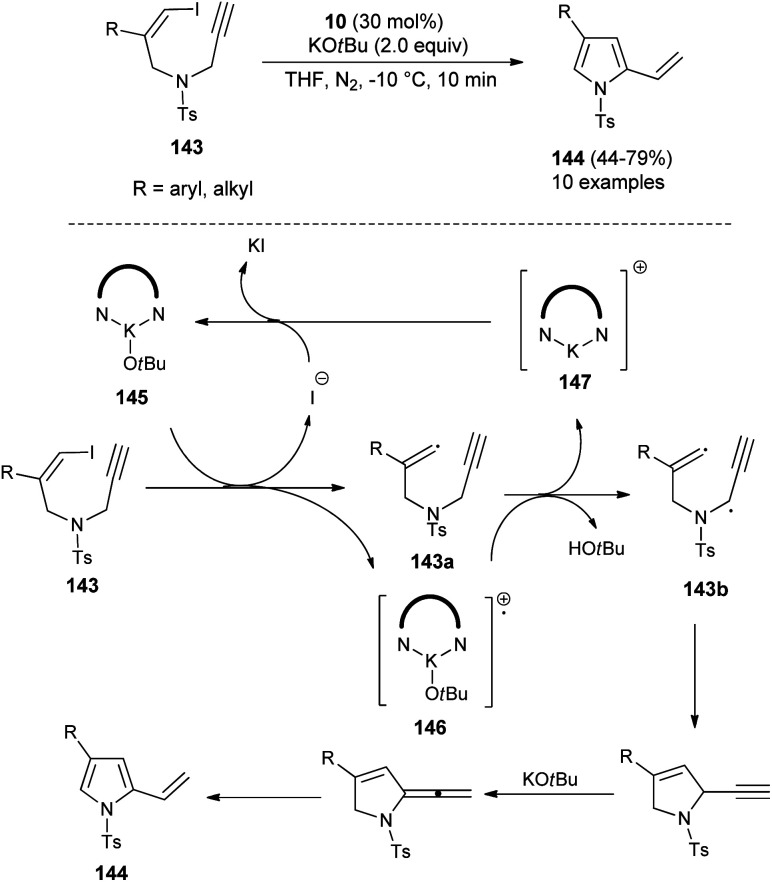
Organocatalytic cycloisomerization reactions to access substituted pyrroles 144.

In 2019, Meng *et al.*^58^ reported that the substrates *Z*-1-iodo-4-*N*-methylbenzenesulfonyl-1,6-enynes 143 bearing vinyl iodide and *N*-propargylamine underwent a cycloisomerization reaction in the presence of organocatalyst 10 and KO*t*Bu in THF at −10 °C, and thereby provided the functionalized pyrroles 144 from non-aromatic to aromatic systems in 44–79% yield within 10 minutes (Scheme 42), although the effect of the organocatalyst in this radical initiation transformation is not clearly described in the report. However, the author suggested a mechanistic pathway for this reaction, which initially involves the homolytic cleavage of the C–I bond of 143 under the influence of complex 145 (10 and *t*-BuOK), leading to the formation of a vinyl radical 143a and complex 146 with subsequent removal of the iodide anion. The abstraction of hydrogen by 146 from the propargylic position furnishes the complex 147 that reconverted into the catalyst 145 by treating with iodide. The double radical 143b undergoes intramolecular cyclization followed by isomerization in the presence of KO*t*Bu and aromatization to yield the corresponding pyrroles 144.

**Scheme 43:**
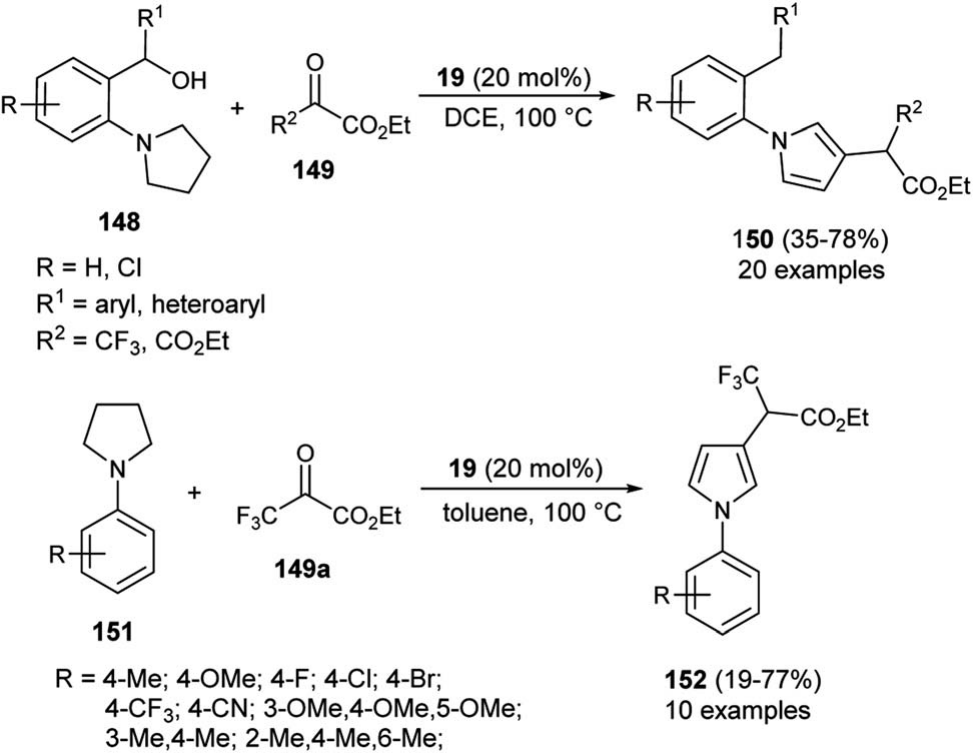
Organocatalytic hydride transfer strategy to access pyrroles 150 & 152.

In 2020, Zhou and his co-workers demonstrated an unprecedented cascade β-functionalization/aromatization reaction of *N*-aryl pyrrolidines for the synthesis of diverse β-substituted aryl pyrroles embedded with trifluoromethyl groups by using 20 mol% of 1,1&-binaphthyl-2,2-diyl hydrogen phosphate 19 as the Brønsted acid catalyst in 1,2-dichloroethane (DCE) as the solvent at 100 °C. The reaction of *N*-aryl pyrrolidines 148 with ketoesters 149 provided the corresponding β-functionalized pyrroles 150 in 35–78% yields (Scheme 43).^59^ This reaction proceeded through the intramolecular [1,5]-hydride transfer (HT) initiated cascade reaction sequence. It is pertinent to note that various halogens present on the aromatic rings and other aromatic rings, such as naphthalene, acenaphthene, biphenyl, furan, and thiophene, were well tolerated with this transformation. They also expanded the methodology for the synthesis of other pyrrole derivatives 152, and the desired product was obtained in 19–77% yield. To obtained the best yield of the product, the solvent system was replaced by toluene instead of DCE, and the transformation was carried out *via* intermolecular HT-initiated β-C(sp^3^)–H functionalization/aromatization sequence.

## 8. Conclusion

In this review, we have summarized the up-to-date advances on the utilization of organocatalysts for the synthesis of various pyrroles over the last decades. On behalf of the appropriate understanding and a convenient presentation, the article is classified according to the two-component synthesis, multicomponent synthesis, multistep synthesis, formal [3 + 2] cycloaddition, synthesis of axially chiral pyrroles, as well as other synthetic strategies. After the renaissance of organocatalysis, the growth in the field of organic synthetic chemistry for the construction of diverse biologically active building blocks in asymmetric, as well as non-asymmetric fashion, has reached an exceptional level in this century. It can be categorized into several activation modes, including amine catalysis, phase-transfer catalysis, hydrogen-bonding catalysis, and others. In sharp contrast devoted towards its development, the synthesis of pyrrole molecules by organocatalytic strategy is limited. However, several metal-free approaches have been discovered, even though they all are not considered organocatalytic routes. Although remarkable results were obtained, the development of very effective and concise organocatalytic methods for the pyrrole synthesis is still desired. We hope these reviewed methods provide fundamental support to design and develope novel synthetic strategies to access these five-membered *N*-heterocycles that could be of interest in medicinal chemistry, material sciences, as well as many branches of chemistry.

## Conflicts of interest

There are no conflicts to declare.

## Supplementary Material
